# MetFrag relaunched: incorporating strategies beyond *in silico* fragmentation

**DOI:** 10.1186/s13321-016-0115-9

**Published:** 2016-01-29

**Authors:** Christoph Ruttkies, Emma L. Schymanski, Sebastian Wolf, Juliane Hollender, Steffen Neumann

**Affiliations:** Leibniz Institute of Plant Biochemistry, Department of Stress and Developmental Biology, Weinberg 3, 06120 Halle, Germany; Eawag: Swiss Federal Institute for Aquatic Science and Technology, Überlandstrasse 133, 8600 Dübendorf, Switzerland; Institute of Biogeochemistry and Pollutant Dynamics, ETH Zürich, 8092 Zürich, Switzerland; R&D NMR Software, Bruker BioSpin GmbH, Silberstreifen, 76287 Rheinstetten, Germany

**Keywords:** Compound identification, *In silico* fragmentation, High resolution mass spectrometry, Metabolomics, Structure elucidation

## Abstract

**Background:**

The *in silico* fragmenter MetFrag, launched in 2010, was one of the first approaches combining compound database searching and fragmentation prediction for small molecule identification from tandem mass spectrometry data. Since then many new approaches have evolved, as has MetFrag itself. This article details the latest developments to MetFrag and its use in small molecule identification since the original publication.

**Results:**

MetFrag has gone through algorithmic and scoring refinements. New features include the retrieval of reference, data source and patent information via ChemSpider and PubChem web services, as well as InChIKey filtering to reduce candidate redundancy due to stereoisomerism. Candidates can be filtered or scored differently based on criteria like occurence of certain elements and/or substructures prior to fragmentation, or presence in so-called “suspect lists”. Retention time information can now be calculated either within MetFrag with a sufficient amount of user-provided retention times, or incorporated separately as “user-defined scores” to be included in candidate ranking. The changes to MetFrag were evaluated on the original dataset as well as a dataset of 473 merged high resolution tandem mass spectra (HR-MS/MS) and compared with another open source *in silico *fragmenter, CFM-ID. Using HR-MS/MS information only, MetFrag2.2 and CFM-ID had 30 and 43 Top 1 ranks, respectively, using PubChem as a database. Including reference and retention information in MetFrag2.2 improved this to 420 and 336 Top 1 ranks with ChemSpider and PubChem (89 and 71 %), respectively, and even up to 343 Top 1 ranks (PubChem) when combining with CFM-ID. The optimal parameters and weights were verified using three additional datasets of 824 merged HR-MS/MS spectra in total. Further examples are given to demonstrate flexibility of the enhanced features.

**Conclusions:**

In many cases additional information is available from the experimental context to add to small molecule identification, which is especially useful where the mass spectrum alone is not sufficient for candidate selection from a large number of candidates. The results achieved with MetFrag2.2 clearly show the benefit of considering this additional information. The new functions greatly enhance the chance of identification success and have been incorporated into a command line interface in a flexible way designed to be integrated into high throughput workflows. Feedback on the command line version of MetFrag2.2 available at http://c-ruttkies.github.io/MetFrag/ is welcome.

**Electronic supplementary material:**

The online version of this article (doi:10.1186/s13321-016-0115-9) contains supplementary material, which is available to authorized users.

## Background

The identification of unknown small molecules from mass spectral data is one of the most commonly-mentioned bottlenecks in several scientific fields, including metabolomic, forensic, environmental, pharmaceutical and medical sciences. Recent developments to high resolution, accurate mass spectrometry coupled with chromatographic separation has revolutionized high-throughput analysis and opened up whole new ranges of substances that can be detected at ever decreasing detection limits. However, where “peak inventories” are reported, the vast majority of the substances or peaks detected in samples typically remain unidentified [1–3]. Although targeted analysis, where a reference standard is available, remains the best way to confirm the identification of a compound, it is no longer possible to have access to reference standards for the 100s–1000s of substances of interest in complex samples. While mass spectral libraries are growing for high accuracy tandem and $\hbox{MS}{^n}$ spectra, the coverage is still relatively small compared with the number of compounds that could potentially be present in typical samples [4, 5]. Thus, for substances without reference standards or not present in the spectral libraries, the challenge of identification still remains. This has spurred activities in computational mass spectrometry, aimed at proposing tentative identifications for the cases where the mass spectrum is not (yet) in a mass spectral library.

The *in silico* fragmenter MetFrag, launched in 2010, was one of the first approaches to address this niche for accurate tandem mass spectra in a fast, combinatorial manner [6]. The MetFrag workflow starts by retrieving candidate structures from the compound databases PubChem [7], ChemSpider [8] or KEGG [9, 10], or accepting the upload of a structure data file (SDF) containing candidates. Candidates are then fragmented using a bond dissociation approach and these fragments are compared with the product ions in the measured mass spectrum to determine which candidates best explain the measured data. The candidate scoring is a function of the mass to charge ratio (*m*/*z*),  intensity and bond dissociation energy (BDE) of the matched peaks, while a limited number of neutral loss rules (5 in total) account for rearrangements [6]. Searching PubChem, the original MetFrag (hereafter termed “MetFrag2010” for readability) achieved a median rank of 8 (with an average of 338 candidates per compound) when restricted to a Feb. 2006 version of PubChem, and 31.5 querying PubChem in 2009 (average of 2508 candidates per compound) on a 102 compound dataset from Hill et al. [11]. As PubChem is now double the size of the 2009 version, the candidate ranking becomes more challenging over time due to the increase in numbers of candidates. Thus, innovations are required to improve performance and efficiency.

Other methods for *in silico* fragmentation are also available. The commercial software Mass Frontier [12] uses rule–based fragmentation prediction based on standard reactions, a comprehensive library of over 100,000 fragmentation rules, or both. The approaches of MetFrag and Mass Frontier are complementary and have been used in combination to support structure elucidation [13, 14], but Mass Frontier does not perform candidate retrieval or scoring by itself. With increasing amounts of data available, machine learning approaches have been used to train models of the fragmentation process. Heinonen et al. [15] introduced FingerID, which uses a support vector machine to learn the mapping between the mass spectra and molecular fingerprints of the candidates. Allen et al. [16] use a stochastic, generative Markov model for the fragmentation. Implemented in CFM-ID (competitive fragment modelling), this can be used to assign fragments to spectra to rank the candidates, but also to predict spectra from structures alone. The MAGMa algorithm [17] includes information from $\hbox{MS}^n$ fragmentation data, but also uses the number of references as an additional scoring term. The latest fragmenter, CSI:FingerID combines fragmentation trees and molecular fingerprinting to achieve up to 39 % Top 1 ranks, outperforming all other fragmenters [18]. The MetFusion [19] approach takes advantage of the availability of spectral data for some compounds and performs a combined query of both MetFrag and MassBank [20], such that the scores of candidates with high chemical similarity to high-scoring reference spectra are increased.

Lessons from recent critical assessment of small molecule identification contests (CASMI) [21, 22], which included many of the above-mentioned algorithms, show that the use of smaller, specific databases greatly improves the chance of obtaining the correct answer ranked highly and that the winners gathered information from many different sources, rather than relying on the *in silico *fragmentation alone. Furthermore, performing candidate selection by molecular formula can risk losing the correct candidate if the formula prediction is not certain, such that an exact mass search can be more appropriate in cases where more than one formula is possible. Despite the progress achieved for *in silico *fragmentation approaches, there are still some fundamental limitations to mass spectrometry that mean that candidate ranking cannot be solved by fragment prediction alone. For example, mass spectra that are dominated by one or only a few fragments (e.g. a water loss) that can be explained by most of the candidates simply do not contain enough information to distinguish candidates. Further examples and limitations are discussed extensively in [4].

The aim of MetFrag2.2 was to incorporate many additional features into the original MetFrag *in silico *fragmenter, considering all the information presented above. Features to explicitly include or exclude combinations of elements and substructures by either filtering or scoring were added. Suspect screening approaches, growing in popularity in environmental analysis [1], were also incorporated to allow users to screen large databases (i.e. PubChem and ChemSpider) while being able to check for candidates present in smaller, more specific databases (e.g. KEGG [9], HMDB [23], STOFF-IDENT [24], MassBank [20] or NORMAN suspects [25]), enabling users to “flag” potential structures of interest. The number of references, data sources and/or patents for a substance are now accessible via PubChem and/or ChemSpider web services, and a PubChem reference score has already been included in the MAGMa web interface [26]. A high number of literature references or patent listings may indicate that the substance is of high use and thus more likely to be found in the environment. Similarly, a higher number of scientific articles for a metabolite could indicate that this has been observed in biological samples before. Reference information has been shown to increase identification “success” in many cases, for example [17, 27, 28], by providing additional information completely independent of the analytical evidence. However, as this information can introduce a bias towards known compounds, this information should be incorporated with caution, depending on the experimental context.

Retention time information is often used for candidate selection in LC/MS. Unlike the retention index (RI) in GC, where the Kovats RI [29] is quite widely applied, there is not yet an established RI per se for LC/MS despite a high interest. Instead, where a reverse phase column is used for the LC method, the octanol–water partitioning coefficient (log *P*) and retention times (RT) of substances can be correlated due to the column properties [30]. The log *P* of the measured standards can be predicted with various software approaches and correlated with the retention times (see e.g. [31] for an overview on different methods). This has already been used in candidate selection (e.g. [13, 32–34]), with various log *P* predictions. The orthogonal information proved useful despite the large errors associated with the predictions (e.g. over 1 log unit or up to several minutes retention time window depending on the LC run length). These are due to uncertainties in log *P* prediction that are common among different prediction implementations when considering a broad range of substances with different (and many) functional groups and ionization behaviour. As the Chemical Development Kit (CDK [35, 36]) offers log *P* calculations, this can be incorporated within MetFrag2.2. Alternative approaches with log *D*, accounting for ionization, or those requiring more extensive calculations (e.g. [37–39]) can be included via a user-defined score, described further below.

This article details the developments and improvements that have been made to MetFrag since the original publication, including a detailed evaluation on several datasets and specific examples to demonstrate the use of MetFrag2.2 in small molecule identification.

## Implementation

### MetFrag architecture

MetFrag2.2 is written in Java and uses the CDK [35] to read, write and process chemical structures. To start, candidates are selected from a compound database based on the neutral monoisotopic precursor mass and a given relative mass deviation (e.g. 229.1089 ± 5 ppm), the neutral molecular formula of the precursor or a set of database-dependent compound accession numbers. Currently, the online databases KEGG [9, 10], PubChem [7] or ChemSpider [8] can be used with MetFrag2.2, as well as offline databases in the form of a structure data file (SDF) or, new to MetFrag2.2, a CSV file that contains structures in the form of InChIs [40] together with their identifiers and other properties. Furthermore, MetFrag2.2 is able to query local compound database systems in MySQL or PostgreSQL, as performed in [41].

MetFrag2010 considered the ion species [M + H]$^{+}$, [M]$^{+}$, [M]$^{-}$ and [M − H]$^{-}$ during candidate retrieval and fragment generation. While the web interface contained an adduct mass adjustment feature, the presence of adducts was not considered in the fragments. MetFrag2.2 can also handle adducts also appearing in the product ions associated with [M + Na]$^+$, [M + K]$^+$, [M + NH$_4$]$^+$ for positive ionization and [M + Cl]$^-$, [M + HCOO]$^-$ and [M + CH$_3$COO]$^-$ for negative ionization. As the candidate retrieval is performed on neutral molecules, the precursor adduct type must still be known beforehand; for high-throughput workflows this information is intended to come from the workflow output.

Additive relative and absolute mass deviation values are used to perform the MS/MS peak matching and can be adjusted according to the instrument type used for MS/MS spectra acquisition. The number of fragmentation steps performed by MetFrag2.2 can be limited by setting the tree depth (default is 2).

The overall score of a given candidate is calculated as shown in Eq. 1.

1 $$\begin{aligned} S_{C_{{{\mathrm{Final}}}}}&= {} \omega _{{{\mathrm{Frag}}}} \cdot S_{C_{{\mathrm{Frag}}}} + \omega _{{\mathrm{RT}}} \cdot S_{C_{{\mathrm{RT}}}} + \omega _{{\mathrm{Refs}}} \cdot S_{C_{{\mathrm{Refs}}}} + \omega _{{\mathrm{Incl}}} \cdot S_{C_{{\mathrm{Incl}}}} \\ \nonumber&\quad +\,\omega _{{\mathrm{Excl}}} \cdot S_{C_{{\mathrm{Excl}}}} + \omega _{{\mathrm{Suspects}}} \cdot S_{C_{{\mathrm{Suspects}}}} + \cdots + \omega _n \cdot S_{C_{{\mathrm{n}}}} \end{aligned}$$

The final candidate score $S_{C_{{\mathrm{Final}}}}$ is the weighted sum of all single scoring terms used, where the weights given by $\omega _i$ specify the contribution of each term. All $S_{C}$ scoring terms used to calculate $S_{C_{{\mathrm{Final}}}}$ are normalized to the maximum value within the candidate result list for a given MS/MS input. The calculation of individual scoring terms are detailed in the subsections below; all terms besides $S_{C_{{\mathrm{Frag}}}}$ are new to MetFrag2.2.

A variety of output options are available. Output SDFs contain all compounds with a structure connection table and all additional information stored in property fields. For the CSV and XLS format, the structures are encoded by SMILES [42] and InChI codes, while an extended XLS option is available that includes images of the compounds and/or fragments. In all cases the compounds are sorted by the calculated score by default.

### *In silico* fragmentation refinements

The *in silico *fragmentation part of MetFrag2.2 has undergone extensive algorithmic and scoring refinements. The fragmentation algorithm still uses a top-down approach, starting with an entire molecular graph and removing each bond successively. However, the generated fragments are now stored more efficiently by using only the indexes of removed bonds and atoms, similar to the MAGMa approach [43]. This not only increases processing speed and decreases memory usage, but still allows the fast calculation of the masses and molecular formulas of each fragment. This makes it possible to process MS/MS spectra with higher tree depths to generate reliable fragments for molecules with complex ring structures with lower CPU and memory requirements. As a result, fragment filters such as the molecular formula duplicate filter used in MetFrag2010 to decrease the number of generated structures were no longer required, their removal reduces the risk of missing a potentially correct fragment. The calculation of the fragmentation score, $S_{C_{{\mathrm{Frag}}}}$, modified from the score given in [6], is shown in Eq. 2 for a given candidate C:

2 $$S_{C_{{\mathrm{Frag}}}} = \sum\limits_{p \in P} \frac{{{{\mathrm{RelMass}}}_p}^\alpha \cdot {{{\mathrm{RelInt}}}_p}^\beta }{\left( \sum \nolimits _{b \in B_f} {{\mathrm{BDE_b}}}\right) ^\gamma }$$

For each peak *p* matching a generated fragment, the relative mass ${{\mathrm{RelMass}}}_p$ and intensity ${{\mathrm{RelInt}}}_p$ as well as the sum of all cleaved bonds *b* of the fragment *f* assigned to *p* are considered. Where more than one fragment could be assigned to *p*, the fragment with the lowest denominator value is considered. In contrast to Eq. 2, the MetFrag2010 scoring used the difference between $1{/}max(w_c)$ and $1{/}max(e) \cdot e_c$, which could lead to negative scores if the BDE penalty was large. The weights $\alpha$, $\beta$ and $\gamma$ were optimized on a smaller subset of spectra from Gerlich and Neumann [19] that was not used further in this work including merged MassBank IPB (PB) and RIKEN (PR) MS/MS spectra and were set to $\alpha = 1.84$, $\beta = 0.59$ and $\gamma = 0.47$. Once $S_{C_{{\mathrm{Frag}}}}$ has been calculated for all candidates within a candidate list, it is normalised so that the highest score is one.

### Compound filters, element and substructure options

The *unconnected compound filter* was already implemented in MetFrag2010 to remove salts and other unconnected substances that could not possibly have the correct neutral mass from the candidate list. InChIKey filtering has now been added to reduce candidate redundancy due to stereoisomerism, as stereoisomers inflate candidate numbers but cannot (usually) be distinguished with MS/MS. The InChIKey filtering is performed using the first block, which encodes the molecular skeleton (or connectivity), but not the stereochemistry. While this is generally reasonable, some tautomers may have differing InChIKey first blocks (see e.g. [40]), such that not all tautomers will be filtered out. The highest-scoring stereoisomers overall with a matching first block are retained.

*Element restrictions* have been added to enhance the specificity of the exact mass search. Three options are available to restrict the elements considered: (a) include *only* the given elements, (b) the given elements have to be present, but other elements can also be present (as long as they are not explicitly excluded) and (c) exclude certain elements. Options (b) and (c) can be used in combination. These filters can be used for example to incorporate isotope information (e.g. Cl, S) that has been detected in the full scan (MS1) data.

*Substructure restrictions* allow the inclusion and exclusion of certain molecular substructures, encoded in SMARTS [44]. Each substructure is searched independently, thus overlapping substructures can also be considered. This option is particularly useful for cases where detailed information about a parent substance is known (e.g. transformation product, metabolite elucidation), or complementary substructure information is available from elsewhere (e.g. MS2Analyzer [45] or other MS classifiers [13]). Candidates containing certain substructures can either be included and/or excluded prior to fragmentation, or scored differently. To calculate a score, the number of matches in the inclusion or exclusion list containing *n* substructures are added per candidate as given in Eq. 3 (where $M_i=1,$ if substructure *i* matches candidate *C* from the given candidate list *L* or 0 otherwise):

3 $$N_{C_{{\mathrm{Match}}}} = {\sum } M_1 + M_2 + \cdots + M_n ; \quad M_i \in \{0,1\}$$

The inclusion ($S_{C_{{\mathrm{Incl}}}}$) and/or exclusion ($S_{C_{{\mathrm{Excl}}}}$) score(s) per candidate are then calcualted as shown in Eq. 4:

4 $$S_{C_{{\mathrm{Incl}}}} = \frac{N_{C_{{\mathrm{Match}}}}}{max_{C^{{\prime }} \in L}\left( N_{{C^{{\prime }}}_{{\mathrm{Match}}}}\right) };\quad S_{C_{{\mathrm{Excl}}}} = \frac{n - N_{C_{{\mathrm{Match}}}}}{max_{{C^{{\prime }}} \in L}\left( n - N_{{C^{{\prime }}}_{{\mathrm{Match}}}}\right) }$$

where $max_{C^{{\prime }} \in L}(N_{{C^{{\prime }}}_{{\mathrm{Match}}}})$ is the maximal value of $N_{C_{{\mathrm{Match}}}}$ within the candidate list and the scores $S_{C_{{\mathrm{Incl}}}}$ or $S_{C_{{\mathrm{Excl}}}}$ are set to 0 when $max_{{C^{{\prime }}} \in L}(N_{{C^{{\prime }}}_{{\mathrm{Match}}}}) = 0$ or $max_{{C^{{\prime }}} \in L}(n - N_{{C^{{\prime }}}_{{\mathrm{Match}}}}) = 0$, respectively.

### Additional substance information

#### Reference and patent information

While the reference and patent information is represented by the placeholder term $\omega _{{\mathrm{Refs}}} \cdot S_{C_{{\mathrm{Refs}}}}$ in Eq. 1, the score can either be composed of several terms or added as a combined term, as described below.

If the query databases is PubChem, the number of patents (PubChemNumberPatents, PNP) and PubMed references (PubChemPubMedCount, PPC) are retrieved for each candidate via the PubChem PUG REST API [46]. These values result in the scoring terms $S_{C_{{\mathrm{PNP}}}}$ and $S_{C_{{\mathrm{PPC}}}}$, which can be weighted individually, or a combined term with either or both parameters. For the latter, first, a cumulative reference term is calculated as shown in Eq. 5, before the PubChem combined reference score ($S_{C_{{\mathrm{PCR}}}}$) is calculated for candidate *C* in candidate list *L* as shown in Eq. 6 for PubChem:

5 $$N_{C_{{\mathrm{{PCR}}}}} = a_1 \cdot {{\mathrm{PNP}}}_C + a_2 \cdot {{\mathrm{PPC}}}_C, \quad a_1, a_2 \in \{0, 1\}$$

6 $$S_{C_{{\mathrm{PCR}}}} = \frac{N_{C_{{\mathrm{{PCR}}}}}}{max_{C^{{\prime }} \in L} N_{C^{{\prime }}_{{{\mathrm{PCR}}}}}}$$

For ChemSpider, five values with reference information can be retrieved using the ChemSpider web services [47]), including the number of data sources (ChemSpiderDataSourceCount, CDC), references (ChemspiderReferenceCount, CRC), PubMed references (ChemSpiderPubMedCount, CPC), Royal Society for Chemistry (RSC) references (ChemSpiderRSCCount, CRSC) and external references (ChemSpiderExternalReferenceCount, CERC). Any combination of these reference sources can be used and weighted individually, yielding the score terms $S_{C_{{\mathrm{CDC}}}}$, $S_{C_{{\mathrm{CRC}}}}$, $S_{C_{{\mathrm{CPC}}}}$, $S_{C_{{\mathrm{CRSC}}}}$ and $S_{C_{{\mathrm{CERC}}}}$. Alternatively, the ChemSpider Combined Reference Scoring term ($S_{C_{{\mathrm{CCR}}}}$) can be calculated, as shown below in Eqs. 7 and 8:

7 $$\begin{aligned} N_{C_{{\mathrm{{CCR}}}}}&= b_1 \cdot {{\mathrm{CRC}}}_C + b_2 \cdot {{\mathrm{CERC}}}_C + b_3 \cdot {{\mathrm{CRSC}}}_C \nonumber \\&\quad + b_4 \cdot {{\mathrm{CPC}}}_C + b_5 \cdot {{\mathrm{CDC}}}_C \quad b_1, b_2, b_3, b_4, b_5 \in \{0, 1\} \end{aligned}$$

8 $$S_{C_{{\mathrm{CCR}}}} = \frac{N_{C_{{\mathrm{{CCR}}}}}}{max_{{C^{{{\prime }}}} \in L}\,N_{C^{{\prime }}_{{{\mathrm{CCR}}}}}}$$

The corresponding command line terms are given in the additional information (see Additional files 1, 2, 3).

#### Suspect lists

Additional lists of substances (so-called “suspect lists”) can be used to screen for the presence of retrieved candidates in alternative databases. The suspect lists are input as a text file containing InChIKeys (one key per line) for fast screening. The first block of the InChIKey is used to determine matches. Example files are available from [25]. This “suspect screening” can be used as an inclusion filter (include only those substances that are in the suspect list) or as an additional scoring term for the ranking of the candidates, yielding the term $\omega _{{\mathrm{Suspects}}} \cdot S_{C_{{\mathrm{Suspects}}}}$ given in Eq. 1.

### Retention time score via log *P*

The retention time (RT) scores offered within MetFrag2.2 are based on the correlation of log *P* and user-provided RT information. The RTs must be associated with sufficient analytical standards measured under the same conditions as the unknown spectrum (a minimum of ten data points are recommended, depending on the distribution over the chromatographic run). By default, the log *P* is calculated using the XlogP algorithm in the CDK library [36, 48, 49]. Alternatively, if PubChem is used as a candidate source, the XLOGP3 value retrieved from PubChem can also be used [50]. The user-provided RTs and their associated log *P* values comprise a training dataset to generate a linear model between RT and the log *P*, shown in Eq. 9, where *a* and *b* are determined using least squares regression:

9 $${{\mathrm{log}}}\,P_{{\mathrm{Unknown}}} = a \cdot {{\mathrm{RT}}}_{{\mathrm{Unknown}}} + b$$

This equation is then used to estimate log $P_{{\mathrm{Unknown}}}$, given the measured RT associated with the unknown spectrum, and compared with log $P_{{\mathrm{C}}}$ calculated for each candidate. It is imperative that the log *P* calculated for each candidate arises from the same source as the log *P* used to build the model in Eq. 9. Lower log *P* deviations result in a higher score for a candidate; the score is calculated using density functions assuming a normal distribution with $\sigma =1.5$ (chosen arbitrarily), as shown in Eq. 10:

10 $$S_{C_{{\mathrm{RT}}}} = \frac{1}{{\sigma \sqrt{2\pi } }} e^{{{ - \left( {|{{\mathrm{log}}}\,P_{{{\mathrm{Unknown}}}}- {{\mathrm{log}}}\,P_{{{\mathrm{C}}}}|} \right) ^2 }/{2\sigma ^2 }}}$$

Alternative log *P* values that are not available within MetFrag2.2 can also be used to establish a model and calculate a different $S_{C_{{\mathrm{RT}}}}$ in a two-step approach. First, MetFrag2.2 can be run either with or without one of the built-in models, so that candidates and all other scores can be obtained. The InChIs or SMILES in the output CSV, or structures in the output SDF can then be used by the user to calculate their own log *P* values. These should be included in the output CSV or SDF using the “UserLogP” tag (or a self-defined alternative) and used as input for MetFrag2.2 with the Local Database option and a RT training file containing retention times and the user log *P*s with the column header matching the tag in the results file. The values *a* and *b* in Eq. 9 are then determined and used to calculate $S_{C_{{\mathrm{RT}}}}$ for the final scoring. Alternative RT models that do not use log *P* should be included as a “user-defined score”, as described below.

### User-defined scoring functions

The final term in Eq. 1, $\omega _n \cdot S_{C_{{\mathrm{n}}}}$, represents the “user-defined scoring function”, which allows users to incorporate any additional information into the final candidate scoring. The MetFrag2.2 output (InChIs, SMILES, SDF) can be used to calculate additional “scores” for the candidates using external methods and these scores can be reimported with the candidates and all other MetFrag2.2 scores in the pipe-separated (|) format for final scoring. The scores and weights are matched from the column headers in the input file and the parameter names added to the score list. The commands are given in a additional table (see Additional files 1, 2, 3), with an example (“terbutylazine and isomers”) below.

## Results and discussion

The changes to MetFrag2.2 were evaluated on several datasets, described in the following. Further examples are given to demonstrate the use of different new features. Unless mentioned otherwise, candidate structures were retrieved from the compound databases PubChem and ChemSpider in June, 2015. If not stated explicitly, the datasets were processed with a relative and absolute fragment mass deviation of 5 ppm and 0.001 Da, respectively. The resulting ranks, if not specified explicitly, correspond to pessimistic ranks, where the worst rank is reported in the case where the correct candidate has the same score as other candidates. Stereoisomers were filtered to keep only the best scored candidate based on the comparison of the first part of the candidates’ InChIKeys. The expected top ranks calculated as in Allen et al. [16], which handles ties of equally scored candidates in a uniformly random manner, are also given when comparing the two *in silico *fragmenters. This demonstrates the effect of equally scored candidates on ranking results.

The datasets from Eawag and UFZ used in this publication arose from the measurement of reference standard collections at Eawag and UFZ, which comprise small molecules of environmental relevance such as pharmaceuticals and pesticides with a wide range of physico-chemical properties and functional groups, and also include several transformation products which typically have lower reference counts. All spectra are publicly available in MassBank.

### *In Silico* fragmentation performance

#### Comparison with MetFrag2010

The merged spectra from 102 compounds published in Hill et al. [11], also used in [6, 19], formed the first evaluation set. The candidate sets from Gerlich and Neumann [19] were used as input for MetFrag2.2 and processed with consistent settings: relative mass deviation of 10 ppm and absolute mass deviation of 0 Da, i.e. no absolute error, for a direct comparison with MetFrag2010. With MetFrag2.2, the median rank improved from 18.5 to 14.5, while the number of correct ranked candidates in the top 1, 3 and 5 improved from 8 to 9, 20 to 24 and 28 to 34, respectively.

#### Baseline performance on Orbitrap XL Dataset

A set of 473 LTQ Orbitrap XL spectra resulting from 359 reference standards formed the second dataset. The spectra were measured at several collision energies with both collision-induced ionization (CID) 35 and higher-energy CID (HCD) 15, 30, 45, 60, 75 and 90 normalized units (see [51] for more details) coupled with liquid chromatography (LC) with a 25 min program on an Xbridge C18 column. The raw files were processed with RMassBank [51, 52], yielding the “EA” records in MassBank. These spectra were merged using the mzClust_hclust function in xcms [53] (parameters eppm = $5 \times 10^{-6}$ and eabs = 0.001 Da) to create peaks with the mean *m*/*z* value and highest (relative) intensity and retained where they contained at least one fragment peak other than the precursor. In total 473 spectra (319 [M + H]$^{+}$and 154 [M − H]$^{-}$) were evaluated with MetFrag2010 using ChemSpider, as well as MetFrag2.2 using either PubChem or ChemSpider. The correct molecular formula was used to retrieve candidates. The results, given in Table 1, show the clear improvement between MetFrag2010 (73 Top 1 ranks with ChemSpider) and MetFrag2.2 (105 top 1 ranks with ChemSpider). This is also indicated by the higher relative ranking positions (RRP) [19] retrieved by MetFrag2.2 where a value of 1 marks the best possible result and 0 the worst possible result. Note that the version used here is 1-RRP as defined in Kerber et al. [54] and Schymanski et al. [55]. The results show that the algorithmic refinements improved the baseline *in silico *fragmentation performance, although it is difficult to tell which of the changes had the greatest influence.

**Table 1:** Comparison of *in silico *fragmentation results for 473 Eawag Orbitrap spectra (formula search)

	MetFrag2010	MetFrag2.2		CFM-ID	MetFrag2.2 + CFM-ID
	ChemSpider	ChemSpider	PubChem	PubChem	PubChem
Pessimistic ranks					
Median rank	8	4	12	11	8
Mean rank	74	38	141	127	85
Mean RRP	0.859	0.894	0.880	0.881	0.901
Top 1 ranks	73 (15 %)	105 (22 %)	30 (6 %)	43 (9 %)	62 (13 %)
Top 5 ranks	202	267	145	170	202
Top 10 ranks	258	320	226	232	276
Expected top ranks					
Top 1 ranks	90 (19 %)	124 (26 %)	43 (9 %)	57 (12 %)	70 (15 %)
Top 5 ranks	218	280	163	193	213
Top 10 ranks	274	329	245	261	288

MetFrag2010 and MetFrag2.2 were compared with the same ChemSpider candidate sets; MetFrag2.2 and CFM-ID with the same PubChem candidate sets. Far right: Best top 1 pessimistic ranks obtained by combining MetFrag2.2 and CFM-ID 2.0 with the weights $\omega _{{\mathrm{Frag}}} = 0.67$ and $\omega _{\mathrm{CFM}\text {-}\mathrm{ID}} = 0.33$. The expected ranks, which partially account for equally scored candidates as calculated in [16], are shown in the lower part of the table

#### Comparison with CFM-ID using Orbitrap XL Dataset

The same dataset of 473 merged spectra and the corresponding PubChem candidate sets were used as input for CFM-ID [16] version 2.0 (“Jaccard”, RDKit 2015.03.1, lpsolve 5.5.2.0, Boost 1.55.0), to form a baseline comparison with an alternative *in silico *fragmenter. The results, given in Table 1, show that CFM-ID generally performed better, indicated by the higher number of correct first ranked candidates (43 vs. 30), top 5 (170 vs. 145), top 10 (232 vs. 226) and a lower median and mean rank of 11 versus 12 and 127 versus 141. The expected ranks, including equal ranked candidates, also implied a better performance of CFM-ID (top 1: 43 vs. 57, top 5: 163 vs. 193, top 10: 245 vs. 261). This was not entirely unexpected as CFM-ID uses a more sophisticated fragmentation approach, but also requires a much longer computation time. For run time analysis, 84 of the 473 queries, selected at random, were processed (single-threaded) with MetFrag2.2 and CFM-ID in parallel on a computer cluster with a maximum of 28 (virtual) computer nodes with 12 CPU cores each. The total run times (system + user runtime, retrieved by linux bash command *time*) were 75 min for MetFrag2.2 and 12,570 min (209.5 h) for CFM-ID. These values represent the runtime on a single CPU core for all 84 queries in series. The average run time per query amounts to 54 s for MetFrag2.2 and 8979 s (150 min) for CFM-ID.

As CFM-ID and MetFrag2.2 use independent *in silico *fragmentation approaches, one can hypothesize that the combination of the approaches should improve the results further. To demonstrate this, the CFM-ID results were incorporated into MetFrag2.2 by introducing an additional scoring term $\omega _{\mathrm{CFM}\text {-}\mathrm{ID}} \cdot S_{C_{\mathrm{CFM}\text {-}\mathrm{ID}}}$, where $S_{C_{{\mathrm{CFM}\text {-}\mathrm{ID}}}}$ defines the normalized CFM-ID probability of candidate *C*. Different contributions of each fragmenter relative to another was determined by randomly drawing 100 combinations of $\omega _{{\mathrm{Frag}}}$ and $\omega _{\mathrm{CFM}\text {-}\mathrm{ID}}$ such that ($\omega _{{\mathrm{Frag}}} + \omega _{\mathrm{CFM}\text {-}\mathrm{ID}} = 1$). The best results, shown in Table 1, were obtained with $\omega _{{\mathrm{Frag}}} = 0.67$ and $\omega _{\mathrm{CFM}\text {-}\mathrm{ID}} = 0.33$, where the change in number 1 ranks with weight is shown in Additional file 4. With this best combination, the number of Top 1 ranks improved from 30 to 61, while the median rank improved to 8. This shows that the combination of independent fragmentation methods can indeed yield valuable improvements to the results, shown again in the next paragraph after including the additional information. Further validation was beyond the scope of the current article, as further improvements could be made by retraining CFM-ID on Orbitrap data, but would be of interest in the future.

### Adding retention time and reference information

#### Parameter selection on Orbitrap XL Dataset

The next stage was to assess the effect of references and retention time information on the MetFrag results. Firstly, each score term (i.e. fragmenter, retention time and/or reference information) was either included or excluded by setting the weight ($\omega _{{\mathrm{Frag}}}, \omega _{{\mathrm{RT}}}, \omega _{{\mathrm{Refs}}}$) to 1 or 0, to assess the impact of the various combinations on the number of correctly-ranked number 1 substances. The results are shown in Table 2. The best result was obtained when all three “score terms” (fragmenter, RT and references) were included in candidate ranking. For PubChem, both RT/log *P* models (CDK XlogP and XLOGP3 from PubChem directly) were assessed and thus two sets of results are reported. The reference information was included using the combined reference scores introduced in Eqs. 6 and 8, where all combinations of the reference values described above (1–2 for PubChem, 1–5 for ChemSpider, i.e. 3 and 31 combinations in total, respectively), were used to form a cumulative total reference term, shown in Eq. 5 for PubChem and Eq. 7 for ChemSpider. The best results were achieved with PubChem when using both patents and PubMed references ($S_{C_{{\mathrm{PNP + PPC}}}}$; $a_1=1$, $a_2=1$), while for ChemSpider using the ReferenceCount, ExternalReferenceCount and the DataSourceCount ($S_{C_\mathrm{CRC + CERC + CDC}}$) proved best, i.e. $b_1 = 1, b_2 = 1, b_3 = 0, b_4 = 0, b_5 = 1$. Table 2 contains the number of Top 1 ranks for each combination of $\omega _{{\mathrm{Frag}}}, \omega _{{\mathrm{RT}}}, \omega _{{\mathrm{Refs}}} \in \{0, 1\}$. The results show clearly that, while references alone result in over 311 top 1 ranks (65 % for PubChem), the addition of both fragmentation and retention time information improves the results further, to 69 % of candidates ranked first (PubChem) and even 87 % of candidates ranked first (ChemSpider). For PubChem the distribution of the number of CombinedReferences (including patents and PubMed references) for the 359 queries of the (unique) correct candidates is shown in Additional file 5.

**Table 2:** PubChem and ChemSpider results (number of pessimistic top 1 ranks) for 473 Eawag Orbitrap spectra

Weight term	Score term	Weights						
$\omega _{{\mathrm{Frag}}}$	$S_{C_{{\mathrm{Frag}}}}$	1	1	1	0	1	0	0
$\omega _{{\mathrm{RT}}}$	$S_{C_{{\mathrm{RT}}}}$	1	1	0	1	0	1	0
$\omega _{{\mathrm{Refs}}}$	$S_{C_{{\mathrm{Refs}}}}$	1	0	1	1	0	0	1

Database	RT source	Top 1 ranks						
PubChem	XLOGP3	325 (69 %)	53	322	315	30	10	311
PubChem	CDK XlogP	326 (69 %)	43	322	316	30	8	311
ChemSpider	CDK XlogP	411 (87 %)	113	411	376	105	41	376

The weights indicate where the score term was included (1) or excluded (0) from the candidate ranking. For PubChem $\omega _{{\mathrm{Refs}}} \cdot S_{C_{{\mathrm{Refs}}}} = \omega _{{\mathrm{Refs}}} \cdot (S_{C_{{\mathrm{PNP + PPC}}}})$; for ChemSpider $S_{C_{{\mathrm{Refs}}}} = S_{C_{{\mathrm{CRC + CERC + CDC}}}}$ only. See text for explanations

**Fig. 1:**
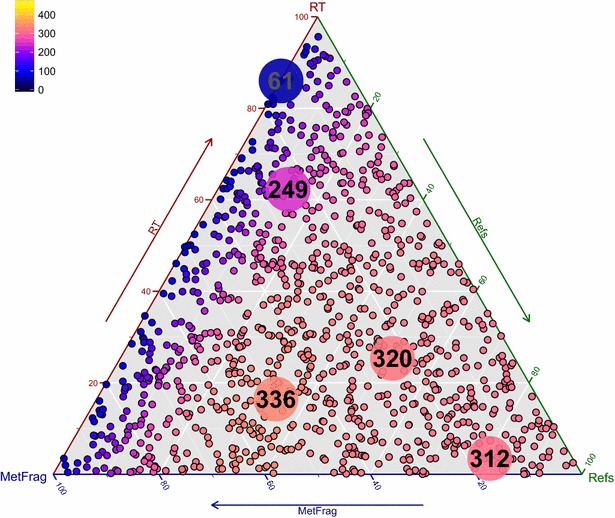
Top 1 ranks with PubChem (XlogP3) on the Orbitrap XL Dataset. The results were obtained with MetFrag formula query and the inclusion of references and retention time. The reference score was calculated with the number of patents (PNP) and PubMed references (PPC). The *larger dots* show the best result (336 number 1 ranks), 75th percentile (320), median (312), 25th percentile (249) and worst result (61). For the best result, the weights were $\omega _{{\mathrm{Frag}}} = 0.50, \omega _{{\mathrm{RT}}} = 0.16$ and $\omega _{{\mathrm{Refs}}} = 0.34$

**Fig. 2:**
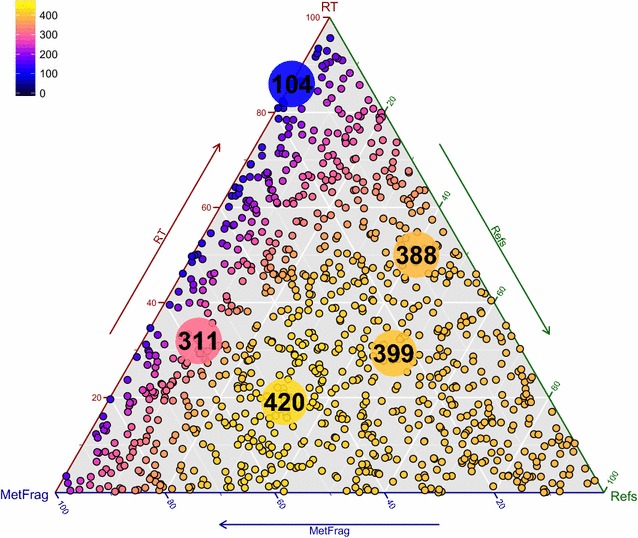
Top 1 ranks with ChemSpider on the Orbitrap XL Dataset. The results were obtained with MetFrag formula query and the inclusion of references and retention time. The reference score was calculated with the ChemSpider reference count (CRC). The *larger dots* show the best result (420), 75th percentile (399), median (388), 25th percentile (311) and worst result (104). The weights for the best result were $\omega _{{\mathrm{Frag}}} = 0.49, \omega _{{\mathrm{RT}}} = 0.19$ and $\omega _{{\mathrm{Refs}}} = 0.32$

Following this, the combination of each scoring term was assessed by randomly drawing 1000 different weight combinations such that ($\omega _{{\mathrm{Frag}}} + \omega _{{\mathrm{RT}}} + \omega _{{\mathrm{Refs}}} = 1$) to determine the optimal relative contributions of each term for the best results. This was performed for all combinations of reference sources (3 for PubChem, 31 for ChemSpider). The best result was obtained again when using both patents and PubMed references for PubChem ($S_{C_{{\mathrm{PNP + PPC}}}}$; $a_1=1$, $a_2=1$), but using only the ReferenceCount ($S_{C_{{\mathrm{CRC}}}}$; $b_1=1$, $b_2=0$, $b_3=0$, $b_4=0$, $b_5=0$) for ChemSpider. The results are summarized in Table 3 (including the weight terms) and shown in Figs. 1 and 2 for PubChem and ChemSpider respectively. These triangle plots show the top 1 candidates for all $\omega _i$ combinations, colour-coded (black—0 % of the correct candidates ranked first, yellow—10 0 % of the correct candidates ranked first) with the $\omega _i$ per category increasing in the direction of the arrow. Each corner is $\omega _i = 1$. The 25th and 75th percentiles are shown to give an idea of the distribution of the ranks. The equivalent plots for the number of top 5 and top 10 ranks are given in Additional files 6, 7, 8 and 9. Although the results from ($\omega _{{\mathrm{Frag}}}$, $\omega _{{\mathrm{RT}}}$, $\omega _{{\mathrm{Refs}}} \in \{0,1\}$) above indicated that the term $S_{C_{{\mathrm{CRC + CERC + CDC}}}}$ yielded the best result for ChemSpider with 411 top 1 ranks, $S_{C_{{\mathrm{CRC}}}}$ yielded 410 top 1 ranks for the same calculations, indicating that there is little difference between the two combinations. Using the randomly-drawn weights, the top 1 ranks improved to 420 (ChemSpider) and 336 (PubChem). This proves without a doubt that the addition of reference and retention time information drastically improves the performance, going from 22 to 89 % top 1 ranks (ChemSpider) and 6.3 to 71 % (PubChem).

**Table 3:** PubChem and ChemSpider results for 473 Eawag orbitrap spectra with formula retrieval, including *in silico *fragmentation, RT and reference information as shown, with the given $\omega _i$ for the highest number of Top 1 ranks

	MetFrag2.2			MetFrag2.2 + CFM-ID
Database	ChemSpider	PubChem	PubChem	PubChem
RT/log *P* Model	CDK XlogP	CDK XlogP	XLOGP3	CDK XlogP
$\omega _{{\mathrm{Frag}}}$ ($S_{C_{{\mathrm{Frag}}}}$)	0.49	0.57	0.50	0.33
$\omega _{{\mathrm{RT}}}$ ($S_{C_{{\mathrm{RT}}}}$)	0.19	0.02	0.16	0.03
$\omega _{{\mathrm{Refs}}}$ ($S_{C_{{\mathrm{Refs}}}}$)	0.32	0.41	0.34	0.35
$\omega _{{\mathrm{CFMID}}}$ ($S_{C_{{\mathrm{CFMID}}}}$)	–	–	–	0.29
Median rank	1	1	1	1
Mean rank	6.5	35	41	18
Mean RRP	0.990	0.977	0.977	0.978
Top 1 ranks	420 (89 %)	336 (71 %)	336 (71 %)	343 (73 %)
Top 5 ranks	447	396	398	411
Top 10 ranks	454	422	414	429

For PubChem $\omega _{{\mathrm{Refs}}} \cdot S_{C_{{\mathrm{Refs}}}} = \omega _{{\mathrm{Refs}}} \cdot (S_{C_{{\mathrm{PNP + PPC}}}})$; for ChemSpider $S_{C_{{\mathrm{Refs}}}} = S_{C_{{\mathrm{CRC}}}}$ only. See text for explanations. Far right: combining CFM-ID results to incorporate complementary fragmentation information

As above, it was interesting to investigate whether the addition of a complementary fragmentation technique, i.e. CFM-ID, would improve the results even further. MetFrag2.2 and CFM-ID were combined with retention time and reference information using 1000 randomly drawn combinations of $\omega _{{\mathrm{Frag}}}$, $\omega _{\mathrm{CFM}\text {-}\mathrm{ID}}$, $\omega _{{\mathrm{RT}}}$ and $\omega _{{\mathrm{PNP + PPC}}}$ such that ($\omega _{{\mathrm{Frag}}} + \omega _{\mathrm{CFM}\text {-}\mathrm{ID}} + \omega _{{\mathrm{RT}}}$ + $\omega _\mathrm{PNP + PPC} = 1$). The results, shown in Table 3, indicate that the PubChem results can be improved further, to 343 top 1 ranks (73 %). This is a drastic improvement from the performance of both original fragmenters alone, with CFM-ID alone yielding between 10 and 12 % top 1 hits (expected rank) in their original publication [16] with an older PubChem, the combination of both fragmenters alone yielding 15 % (expected rank) here. These combined results are also drastically better than the latest *in silico *fragmentation results just published for CSI:FingerID. Dührkop et al. [18] investigated each individual fragmenter currently available and compared the results with the CSI:FingerID. Despite using different data and settings to those here, their results on the Agilent dataset indicated that MetFrag2010 and CFM-ID achieved 9 and 12 % top 1 (expected) ranks, which are reasonably comparable with the results presented above. FingerID [15] achieved 19.6 %, while CSI:FingerID achieved 39 % top 1 results, which is a dramatic improvement over the other fragmenters. Since the external information boosted the top 1 ranks to 73 % for MetFrag2.2 plus CFM-ID, one could speculate that the combination of CSI:FingerID, MetFrag2.2 and CFM-ID would result in an even greater performance.

#### Cross-evaluation on additional datasets

As the RT and reference scores are very subjective to experimental context, MetFrag2.2 now contains so many tuneable parameters that it will be beneficial to users when a few default cases are suggested. Thus, once the optimal reference source combinations were determined as described above, alternative datasets were used to re-determine the optimal weights $\omega _{{\mathrm{Frag}}}$, $\omega _{{\mathrm{RT}}}$ and $\omega _{{\mathrm{Refs}}}$ to investigate the variation over different datasets. Three sufficiently large datasets available on MassBank contained good quality MS/MS and RT data, all processed with RMassBank [51].

*UF dataset:* A susbset of the 2758 UFZ Orbitrap XL records were acquired on an Kinetex Core-Shell C18 column from Phenomenex with a 40 min chromatographic program (all others were direct infusion experiments). These MS/MS spectra, arising from [M + H]$^{+}$ and [M − H]$^{-}$ precursors, were recorded at four collision energies: CID 35 and 55 as well as HCD 50 and 80. These spectra were merged and processed as described above for the Orbitrap XL dataset, resulting in 225 merged spectra (“UF” dataset) from 195 substances (184 [M + H]$^{+}$ and 41 [M $-$ H]$^{-}$).

*EQex and EQxPlus datasets:* Two additional Eawag datasets were also available. The “EQex” dataset, measured on a Q Exactive Orbitrap, contained MS/MS spectra associated with [M + H]$^{+}$ and [M − H]$^{-}$ precursors recorded at six different collision energies (HCD 15, 30, 45, 60, 75 and 90). The “EQExPlus” dataset, measured on a Q Exactive Plus Orbitrap, contained MS/MS spectra associated with [M + H]$^{+}$ and [M − H]$^{-}$ precursors recorded at nine different collision energies (HCD 15, 30, 45, 60, 75, 90, 120, 150, 180).

Both datasets were acquired using the same LC set-up as the other Eawag dataset. The MS/MS from these two datasets were merged as above to yield 294 merged spectra from 204 compounds (195 [M $+$ H]$^{+}$ and 94 [M − H]$^{-}$) for the “EQEx” dataset and 314 merged spectra from 232 compounds (219 [M + H]$^{+}$ and 91 [M − H]$^{-}$) for the “EQExPlus” dataset. There was a very small overlap between the different Eawag datasets (5, 2 and 2 substance overlap between EA and EQEx, EA and EQExPlus and EQEx and EQExPlus, respectively).

The overlap between the UFZ and Eawag datasets was larger, with 97, 16 and 21 substances in common between the UFZ and EA, EQEx and EQExPlus datasets, respectively. The overlap was determined using the first block of the InChIKey. As the spectral and retention time data for the substances in the individual datasets were processed independently with different collision energies and ionization modes, none of the overlapping substances were removed from the datasets. The retention times extracted from the MassBank records per substance were used to establish the RT–log *P* model (see Eq. 9) for each dataset independently based on a tenfold cross-validation.

The influence of the different parameters was assessed for each dataset by setting $\omega _{{\mathrm{Frag}}}, \omega _{{\mathrm{RT}}}$ and $\omega _{{\mathrm{Refs}}}$ to either 0 or 1 again; these results are presented in Table 4. As above, the performance improved from between 2 and 9 % of the candidates ranked first using fragmentation alone, through to 64–82 % ranked first when all $\omega _x$ were weighted equally, although the results varied quite dramatically between the datasets. The 473 spectrum dataset used above thus fell within this range.

**Table 4:** Results (Top 1, 5 and 10 ranks) using PubChem formula queries on three additional datasets

Weight term	Score Term	Weights						
$\omega _{{\mathrm{Frag}}}$	$S_{C_{{\mathrm{Frag}}}}$	1	1	1	0	1	0	0
$\omega _{{\mathrm{RTs}}}$	$S_{C_{{\mathrm{RT}}}}$	1	1	0	1	0	1	0
$\omega _{{\mathrm{Refs}}}$	$S_{C_{{\mathrm{Refs}}}}$	1	0	1	1	0	0	1

Dataset	Metric	Ranks						
UF (n = 225)	Top 1 ranks	164 (73 %)	9	163	159	3	2	157
UF (n = 225)	Top 5 ranks	186 (83 %)	48	189	189	36	13	199
UF (n = 225)	Top 10 ranks	191 (53 %)	77	196	192	61	25	204
EQex (n = 289)	Top 1 ranks	235 (81 %)	33	232	230	26	11	223
EQex (n = 289)	Top 5 ranks	263 (91 %)	87	260	258	88	38	276
EQex (n = 289)	Top 10 ranks	270 (93 %)	132	269	263	139	55	280
EQexPlus (n = 310)	Top 1 ranks	190 (61 %)	32	183	182	21	8	181
EQexPlus (n = 310)	Top 5 ranks	238 (77 %)	84	246	238	83	28	243
EQexPlus (n = 310)	Top 10 ranks	254 (82 %)	115	258	247	121	37	256

The weights indicate where ranking parameters were included (1) or excluded (0) from the candidate ranking. Retention time score calculation was performed using the XLOGP3 values of PubChem. $\omega _{{\mathrm{Refs}}} \cdot S_{C_{{\mathrm{Refs}}}} = \omega _{{\mathrm{Refs}}} \cdot S_{C_{{\mathrm{PNP + PPC}}}}$. See text for explanations

Similarly, the optimization of $\omega _{{\mathrm{Frag}}}, \omega _{{\mathrm{RT}}}$ and $\omega _{{\mathrm{Refs}}}$ was performed again for each dataset independently using the 1000 randomly-drawn weights. The results are presented in Table 5 and show that the percentage of top 1 ranks varies widely between the datasets, from 63 to 82 %; the original dataset falls in the middle with 71 %. The results in Table 5 also show that the suggested relative weights to one another remain consistent enough to enable default parameter suggestion, with $\omega _{{\mathrm{Frag}}} \approx 0.5, \omega _{{\mathrm{RT}}} \approx 0.2$ and $\omega _{{\mathrm{Refs}}} \approx 0.3$. All results for the number of top 1 ranks for the three additional datasets are shown in Additional files 10, 11 and 12.

**Table 5:** Best Top 1 rank results on three additional datasets using PubChem formula queries including *in silico *fragmentation, RT and reference information as shown, with the given $\omega _i$

Dataset	MetFrag2.2		
	UFZ (n = 225)	EQex (n = 289)	EQexPlus (n = 310)
$\omega _{{\mathrm{Frag}}}$ ($S_{C_{{\mathrm{Frag}}}}$)	0.40	0.38	0.61
$\omega _{{\mathrm{RT}}}$ ($S_{C_{{\mathrm{RT}}}}$)	0.23	0.27	0.11
$\omega _{{\mathrm{Refs}}}$ ($S_{C_{{\mathrm{Refs}}}}$)	0.37	0.35	0.28
Median rank	1	1	1
Mean rank	58.0	14.6	46.2
Mean RRP	0.972	0.981	0.976
Top 1 ranks	165 (73 %)	236 (82 %)	196 (63 %)
Top 5 ranks	188	261	233
Top 10 ranks	191	268	247

Retention time score calculation was performed using the XLOGP3 values of PubChem. $\omega _{{\mathrm{Refs}}} \cdot S_{C_{{\mathrm{Refs}}}} = \omega _{{\mathrm{Refs}}} \cdot S_{C_{{\mathrm{PNP + PPC}}}}$. See text for explanations

### Specific examples

As the additional features are more difficult to evaluate using large datasets, individual examples are presented below to demonstrate the flexibility of MetFrag2.2 command line (CL), with the corresponding commands give in a different font. Lists of the available parameters are given in Additional files 1, 2 and 3. These examples serve to show how MetFrag2.2 can also be adjusted by the user to explore individual cases in greater detail than during e.g. a high-throughput screening.

#### Gathering evidence for unknown 199.0428

During the NORMAN Collaborative Non-target Screening Trial [1], a tentatively identified non-target substance of *m*/*z* [M − H]$^{-}$ 199.0431 was reported by one participant as mesitylenesulfonic acid (ChemSpider ID (CSID) 69438, formula $\hbox{C}_9\hbox{H}_{12}\hbox{O}_3\hbox{S}$, neutral monoisotopic mass 200.0507) or isomer. The same unknown was detected in the same sample measured at a second institute, where the standard of mesitylenesulfonic acid was available. Although the retention time was plausible (5.96 min), comparing the MS/MS spectra clearly disproved the proposed identification, with many fragments from the unknown absent in the standard spectrum. Thus, MetFrag2.2 was used to investigate other possibilities.

Firstly, the following parameter combination was used, taking the unknown MS/MS peak list from the second participant: ChemSpider exact mass search, fragment error 0.001 Da + 5 ppm, tree depth 2, unconnected compound and InChIKey filter, filter included elements = C, S (as isotope signals were detected in the full scan), experimental RT = 6.20 min, an RT training set of 355 InChIs and RTs measured on the same system and score weights of 1 (fragmenter and RT score) and 0.25 each for four ChemSpider reference sources. This yielded 134 candidates with four different formulas ($\hbox{C}_9\hbox{H}_{12}\hbox{O}_3\hbox{S}, {\hbox{C}_8\hbox{H}_{16}\hbox{S}\hbox{Si}_2}, {\hbox{C}_7\hbox{H}_{13}\hbox{BO}_2\hbox{SSi}}, {\hbox{C}_7\hbox{H}_{10}\hbox{N}_3\hbox{O}_2\hbox{S}}$), all fulfiling the element filter (C, S). $S_{C_{{\mathrm{Final}}}}$ ranged from 0.70 to 2.12, where several candidates had high numbers of references and similar number of peaks explained. Three candidates are shown in Table 6, along with a summary of the information retrieved. The clear top match, ethyl *p*-toluenesulfonate (CSID 6386, shown to the left) was unlikely to be correct, as the MS/MS contained no evidence of an ethyl loss and also had a clear fragment peak at *m*/*z* 79.9556, corresponding with an $\hbox{SO}_3\hbox{H}$ group (thus eliminating alkyl sulfonates from consideration).

**Table 6:** Top MetFrag2.2 candidates for unknown at *m*/*z* 199.0428 with different settings

CSID	6386	69438	6388
			
Original results (134 candidates)			
Rank (n = 134)	1	6	90
#Peaks explained	5	5	5
CDK log $P/S_{C_{{\mathrm{RT}}}}$	1.44/0.167	1.50/0.161	2.02/0.107
$\sum S_{C_{{\mathrm{Refs}}}}$	$94+15+7+70=186$	$179+1+0+40=220$	$32+0+0+21=53$
Substructure interpretation			
Included	S(=O)(=O)O	S(=O)(=O)O	CCc1ccc(cc1)S(=O)(=O)O
Excluded	–	S(=O)(=O)OC	–
Comment	No ethyl loss in MS/MS	Disproven via standard	Present in suspect list

Structures overlaid with the included substructure were generated with AMBIT [57]. See text for details

MetFrag2.2 was run again with the SMARTS substructure inclusion filter, which resulted in 31 candidates but with the same top matching structure. However, adding the SMARTS S(=O)(=O)OC to the exclusion list eliminates the alkyl sulfonate species and resulted in 18 candidates, where the top candidate was now the originally proposed (and rejected) identification mesitylenesulfonic acid, shown in the middle of Table 6. The next matches were substitution isomers. Referring to the MS/MS again, another large peak was present at *m*/*z* 183.0115, which is often observed in surfactant spectra corresponding with a *p*-ethyl benzenesulfonic acid moiety. Running MetFrag2.2 again with a substructure inclusion of CCc1ccc(cc1)S(=O)(=O)O yielded only two candidates, 4-isopropylbenzenesulfonic acid ($S_{C_{{\mathrm{Final}}}} = 2.5,$ CSID 6388), shown to the right in Table 6 and 4-propylbenzenesulfonic acid ($S_{C_{{\mathrm{Final}}}} = 2.0,$ CSID 5506213).

To check the relevance of the proposed candidates in an environmental sample, a “suspect screening” was performed. The STOFF-IDENT database [24] contains over 8000 substances including those in high volume production and use in Europe registered under the European REACH (Registration, Evaluation, Authorisation and Restriction of CHemicals) Legislation. The STOFF-IDENT contents were downloaded (February 2015) and the SMILES were converted to InChIKeys using OpenBabel and given as input to MetFrag as a suspect list. Of the 134 original candidates, only one, 4-isopropylbenzenesulfonic acid, was tagged as being present in the STOFF-IDENT database. This gives additional evidence that indeed 4-isopropylbenzenesulfonic acid is the substance behind the unknown spectrum, however it has not been possible to confirm this identification at this stage due to the lack of a sufficiently pure reference standard.

#### Terbutylazine and isobars

The example of terbutylazine (CSID 20848, see Table 7) shows how MetFrag2.2 can help in gathering the evidence supporting the identification of isobaric substances. Terbutylazine and secbutylazine (CSID 22172) often co-elute in generic non-target chromatographic methods and have very similar fragmentation patterns, but can usually be distinguished from the other common triazine isobars propazine (CSID 4768) and triethazine (CSID 15157) via MS/MS information. However, during the NORMAN non-target screening collaborative trial [1], all four substances were reported as potential matches for the same mass, showing clearly the danger of suspect screening based only on exact mass. For this example, the merged [M + H]$^{+}$MS/MS spectrum of terbutylazine from the EA dataset above (EA02840X) was used as a peak list to run MetFrag2.2, as the correct answer is clear with a reference spectrum. Table 7 shows the data for the four substances mentioned above plus the top match based on fragmentation data alone, *N*-butyl-6-chloro-*N*$^{\prime }$-ethyl-1,3,5-triazine-2,4-diamine (CSID 4954587, given the synonym “*n*Butylazine” hereafter to save space). ChemSpider was used to perform an exact mass search, resulting in a total of 112 structures (data from only five are shown). Five scores were used, all with weight 1: FragmenterScore, ChemSpiderReferenceCount, RetentionTimeScore, SuspectListsScore and SmartsSubstructureInclusionScore. To show the inclusion of external log *P* calculations, ChemAxon JChem for Excel [56] was used to predict log *P* and log *D* at pH 6.8 (the pH of the chromatographic program used) for a training dataset of the 810 substances in the Eawag database on MassBank. The log *P* and log *D* predictions were then performed externally for all MetFrag candidates on the dominant tautomeric species and added to the MetFrag CSV file for final scoring. The scores, shown in Table 7, showed that different candidates were the best match for different categories, indicated in italics. The candidates are ordered by the number of references. As above, STOFF-IDENT was used as a suspect list and all four of the substances reported by trial participants were indeed in STOFF-IDENT. However, Table 7 clearly shows that two can be eliminated using $S_{C_{{\mathrm{Frag}}}}$ and substructure matches (as the MS/MS clearly displays the loss of a ${\hbox{C}_2\hbox{H}_5}$ and $\hbox{C}_4\hbox{H}_9$ group, indicating these are likey attached to a heteroatom, in this case N). Although secbutylazine is scored lower than terbutylazine, the reference count is the main influence here and both substances could be present in an environmental sample—depending on the context.

**Table 7:** Summary of MetFrag2.2 results for terbutylazine and four isobars

Name	Terbutylazine	Propazine	Secbutylazine	Triethazine	*n*Butylazine^a^
CSID	20848	4768	22172	15157	4954587
					
$S_{C_{{\mathrm{Frag}}}}$	0.958	0.765	0.997	0.653	*1.0*
#Peaks explained	11/15	10/15	12/15	8/15	*12/15*
$S_{C_{{\mathrm{CSRefs}}}}$	*286*	204	56	45	4
ChemAxon log *P*	1.65	2.75	2.28	1.11	2.31
$S_{C_{{\mathrm{RT}}}}$ log *P*	0.159	*0.256*	0.223	0.103	0.225
ChemAxon log *D*	1.63	2.75	2.19	0.97	2.23
$S_{C_{{\mathrm{RT}}}}$ log *D*	0.249	0.247	*0.266*	0.192	0.266
Suspect hit	*1*	*1*	*1*	*1*	0
Substructure hits	*2*	0	*2*	1	*2*
Matches	NC(C)(C)C	–	NC(C)CC	N[CH$_{2}$][CH$_{3}$]	NCCCC
	N[CH$_{2}$][CH$_{3}$]		N[CH$_{2}$][CH$_{3}$]		N[CH$_{2}$][CH$_{3}$]
$S_{C_{{\mathrm{Final}}}}$ (log *P*)	*4.22*	3.43	3.69	2.53	2.52
$S_{C_{{\mathrm{Final}}}}$ (log *D*)	*4.56*	3.41	3.85	2.87	2.68
Comment	Correct substance	No longer in use	Can co-elute with 20848		

The predicted log *P* and log *D* from the retention time was 3.17 and 2.18 using a training set of 810 substances calculated externally with ChemAxon and added to MetFrag2.2 via the UserLogP option. Included substructure SMARTS were N[CH$_{2}$][CH$_{3}$], NCCCC, NC(C)CC, NC(C)(C)C

$^\mathrm{a}$Name synonym assigned for space reasons. The values in italics indicates the best result per category. Structures overlaid with the included substructure were generated with AMBIT [57]. See text for details and weights

The large dataset evaluations show that MetFrag2.2 is suitable for high-throughput workflows, with a relatively quick runtime. On the other hand, the detailed examples shows how the various features of MetFrag2.2 can be used to investigate the top candidates in more detail and enhance the interpretation of the results, including the inclusion of external RT/log *P* and/or log *D* information that cannot be calculated within MetFrag2.2 (e.g. due to license restrictions, as in the case of ChemAxon).

## Conclusions

In many cases additional information is available and needed from the experimental context to complement small molecule identification, especially where the mass spectrum alone is not sufficient for candidate selection from a large number of candidates. The results for MetFrag2.2 clearly show the benefit of considering this additional information, with a tenfold improvement compared with MetFrag2.2 fragmentation information alone. The flexibility of the new features in addition to the ability to add user-defined scores means that MetFrag2.2 is ideally suited to high-throughput workflows, but can also be used to perform individual elucidation efforts in greater detail. The ability to incorporate CFM-ID as an additional scoring function shows the potential to improve these results further using complementary *in silico *fragmentation approaches. The parameter files including the spectral data, the candidate, result and ranking files of the used EA, UF, EQEx, EQExPlus and HILL datasets are available at http://msbi.ipb-halle.de/download/CHIN-D-15-00088/ and can be downloaded as ZIP archives. Feedback on the command line version available at http://c-ruttkies.github.io/MetFrag/ is welcome. The new functions greatly reduce the burden on users to collect and merge ever increasing amounts of information available for substances present in different compound databases, thus enabling them to consider much more evidence during their screening efforts.

## Availability and requirements

- Project name: MetFrag2.2;
- Project home page: http://c-ruttkies.github.io/MetFrag/;
- Operating system(s): Platform independent;
- Programming language: Java;
- Other requirements: Java ≥1.6, Apache Maven ≥3.0.4 (for developers);
- License: GNU LGPL version 2.1 or later;
- Any restrictions to use by non-academics: none.

## Additional files

10.1007/s10898-015-0115-8 MetFrag2.2 Command Line (CL) general parameters.
10.1007/s10898-015-0115-8 MetFrag2.2 CL local database parameters (*MySQL*, *PostgresSQL*) 
10.1007/s10898-015-0115-8 MetFrag2.2 CL - Different Scoring terms (MetFragScoreTypes) available for online databases used by MetFrag All or a subset of these values can also be used as a total with CombinedReferenceScore (Table in Additional file 1).
10.1007/s10898-015-0115-8 Top 1 ranks of MetFrag2.2. combined with CFM--ID This figure shows the distribution of the number of top 1 ranks with different weights (100 drawn randomly between 0 and 1) for MetFrag2.2 and CFM--ID. Lightestyellow dot marks the maximum, 62 top 1 ranks at _MetFrag_ = 0.67 and _CFM-ID_ = 0.33. The red dot at the right marks the minimum, 36 top 1 ranks at _MetFrag_ = 0.997 and _CFM-ID_ = 0.003. The most left dot marks 49 top 1 ranks at _MetFrag_ = 0.02 and _CFM-ID_ = 0.98.
10.1007/s10898-015-0115-8 Number of patents and PubMed references shown as CombinedReferences retrieved from PubChem for the Orbitrap XL dataset This figure shows the distribution of the number of references and patents for all candidates (marked by black dots) retrieved from PubChem for the 359 (unqiue) correct candidates (marked with green line) and the additional (wrong) candidates retrieved for each query. The queries are sorted by the number of CombinedReferences for the correct candidate, respectively. The intensity of the black dots indicate the number of candidates which overlap at that position.
10.1007/s10898-015-0115-8 Top 5 ranks with PubChem (XlogP3) on the Orbitrap XL Dataset The results were obtained with MetFrag2.2 formula query and the inclusion of patents, references and retention time. Each small dot shows the number of first ranks with a given combination of weights. The larger dots show the best result (402 in the top 5), 90^*th*^ percentile (386), median (375), 10^*th*^ percentile (325) and worst result (145).
10.1007/s10898-015-0115-8 Top 5 ranks with ChemSpider on the Orbitrap XL Dataset The results were obtained with MetFrag2.2 formula query and the inclusion of references and retention time. Each small dot shows the number of first ranks with a given combination of weights. The larger dots show the best result (463 in the top 5), 90^*th*^ percentile (452), median (440), 10^*th*^ percentile (385) and worst result (195).
10.1007/s10898-015-0115-8 Top 10 ranks with PubChem (XlogP3) on the Orbitrap XL Dataset The results were obtained with MetFrag2.2 formula query and the inclusion of patents, references and retention time. Each small dot shows the number of first ranks with a given combination of weights. Each small dot shows the number of first ranks with a given combination of weights. The larger dots show the best result (422 in the top 10), 90^*th*^ percentile (406), median (391), 10^*th*^ percentile (351) and worst result (187).
10.1007/s10898-015-0115-8 Top 10 ranks with ChemSpider on the Orbitrap XL Dataset The results were obtained with MetFrag2.2 formula query and the inclusion of references and retention time. Each small dot shows the number of first ranks with a given combination of weights. The larger dots show the best result (471 in the top 10), 90^*th*^ percentile (460), median (450), 10^*th*^ percentile (404) and worst result (223).
10.1007/s10898-015-0115-8 Top 1 ranks with PubChem (XlogP3) on the UFZ dataset The results were obtained with MetFrag2.2 formula query and the inclusion of patents, references and retention time. Each small dot shows the number of first ranks with a given combination of weights. The larger dots show the best result (165 in the top 1), 90^*th*^ percentile (159), median (156), 10^*th*^ percentile (112) and worst result (11).
10.1007/s10898-015-0115-8 Top 1 ranks with PubChem (XlogP3) on the EQex dataset The results were obtained with MetFrag2.2 formula query and the inclusion of patents, references and retention time. Each small dot shows the number of first ranks with a given combination of weights. The larger dots show the best result (236 in the top 1), 90^*th*^ percentile (230), median (225), 10^*th*^ percentile (162) and worst result (29).
10.1007/s10898-015-0115-8 Top 1 ranks with PubChem (XlogP3) on the EQexPlus dataset The results were obtained with MetFrag2.2 formula query and the inclusion of patents, references and retention time. Each small dot shows the number of first ranks with a given combination of weights. The larger dots show the best result (196 in the top 1), 90^*th*^ percentile (184), median (181), 10^*th*^ percentile (142) and worst result (28).

## References

[1] Schymanski EL, Singer HP, Slobodnik J, Ipolyi IM, Oswald P, Krauss M, Schulze T, Haglund P, Letzel T, Grosse S (2015). Non-target screening with high-resolution mass spectrometry: critical review using a collaborative trial on water analysis. Anal Bioanal Chem.

[2] Hug C, Ulrich N, Schulze T, Brack W, Krauss M (2014). Identification of novel micropollutants in wastewater by a combination of suspect and nontarget screening. Environ Pollut.

[3] Schymanski EL, Singer HP, Longrée P, Loos M, Ruff M, Stravs MA, Ripollés Vidal C, Hollender J (2014). Strategies to characterize polar organic contamination in wastewater: exploring the capability of high resolution mass spectrometry. Environ Sci Technol.

[4] Stein S (2012). Mass spectral reference libraries: an ever-expanding resource for chemical identification. Anal Chem.

[5] Vinaixa M, Schymanski EL, Neumann S, Navarro M, Salek RM, Yanes O (2015) Mass spectral databases for LC/MS and GC/MS-based metabolomics: state of the field and future prospects. Trends Anal Chem (TrAC). doi:10.1016/j.trac.2015.09.005

[6] Wolf S, Schmidt S, Müller-Hannemann M, Neumann S (2010). In silico fragmentation for computer assisted identification of metabolite mass spectra. BMC Bioinform.

[7] National Center for Biotechnology Information (2016) PubChem Database. https://pubchem.ncbi.nlm.nih.gov/search/search.cgi. Accessed 14 Jan 2016

[8] Royal Society of Chemistry (2016) ChemSpider. http://www.chemspider.com/

[9] Kanehisa M, Goto S (2000). KEGG: kyoto encyclopedia of genes and genomes. Nucleic Acids Res.

[10] Kanehisa M, Goto S, Hattori M, Aoki-Kinoshita KF, Itoh M, Kawashima S, Katayama T, Araki M, Hirakawa M (2006). From genomics to chemical genomics: new developments in KEGG. Nucleic Acids Res.

[11] Hill DW, Kertesz TM, Fontaine D, Friedman R, Grant DF (2008). Mass spectral metabonomics beyond elemental formula: chemical database querying by matching experimental with computational fragmentation spectra. Anal Chem.

[12] HighChem Ltd. (2015) Mass Frontier v. 7. HighChem Ltd., Bratislava

[13] Schymanski EL, Gallampois CMJ, Krauss M, Meringer M, Neumann S, Schulze T, Wolf S, Brack W (2012). Consensus structure elucidation combining GC/EI–MS, structure generation, and calculated properties. Anal Chem.

[14] Chiaia-Hernandez AC, Schymanski EL, Kumar P, Singer HP, Hollender J (2014). Suspect and nontarget screening approaches to identify organic contaminant records in lake sediments. Anal Bioanal Chem.

[15] Heinonen M, Shen H, Zamboni N, Rousu J (2012). Metabolite identification and molecular fingerprint prediction through machine learning. Bioinformatics.

[16] Allen F, Greiner R, Wishart D (2015). Competitive fragmentation modeling of ESI–MS/MS spectra for putative metabolite identification. Metabolomics.

[17] Ridder L, van der Hooft JJJ, Verhoeven S (2014). Automatic compound annotation from mass spectrometry data using MAGMa. Mass Spectrom.

[18] Dührkop K, Shen H, Meusel M, Rousu J, Böcker S (2015). Searching molecular structure databases with tandem mass spectra using CSI:FingerID. Proc Natl Acad Sci.

[19] Gerlich M, Neumann S (2013). MetFusion: integration of compound identification strategies. J Mass Spectrom.

[20] Horai H, Arita M, Kanaya S, Nihei Y, Ikeda T, Suwa K, Ojima Y, Tanaka K, Tanaka S, Aoshima K, Oda Y, Kakazu Y, Kusano M, Tohge T, Matsuda F, Sawada Y, Hirai MY, Nakanishi H, Ikeda K, Akimoto N, Maoka T, Takahashi H, Ara T, Sakurai N, Suzuki H, Shibata D, Neumann S, Iida T, Tanaka K, Funatsu K, Matsuura F, Soga T, Taguchi R, Saito K, Nishioka T (2010). MassBank: a public repository for sharing mass spectral data for life sciences. J Mass Spectrom.

[21] Kasama T, Kinumi T, Makabe H, Matsuda F, Miura D, Miyashita M, Nakamura T, Tanaka K, Yamamoto A, Nishioka T (2014). Winners of CASMI2013: automated tools and challenge data. Mass Spectrom.

[22] Schymanski EL, Neumann S (2013) CASMI: and the winner is $$\ldots$$ Metabolites 3(2):412–439 10.3390/metabo3020412 PMC3901266 24957999

[23] Wishart DS, Jewison T, Guo AC, Wilson M, Knox C, Liu Y, Djoumbou Y, Mandal R, Aziat F, Dong E (2013). HMDB 3.0—the human metabolome database in 2013. Nucleic Acids Res.

[24] LfU: Bayerisches Landesamt für Umwelt (2016) STOFF-IDENT (login required). http://bb-x-stoffident.hswt.de/. Accessed 14 Jan 2016

[25] NORMAN Association (2016) NORMAN Suspect List Exchange. http://www.norman-network.com/?q=node/236. Accessed 14 Jan 2016

[26] Netherlands eScience Center (2016) MAGMa Web Interface. http://www.emetabolomics.org/magma. Accessed 14 Jan 2016

[27] Little J, Cleven C, Brown S (2011). Identification of known unknown utilizing accurate mass data and chemical abstracts service databases. J Am Soc Mass Spectrom.

[28] Little J, Williams A, Pshenichnov A, Tkachenko V (2012). Identification of known unknowns utilizing accurate mass data and ChemSpider. J Am Soc Mass Spectrom.

[29] Kováts E (1958). Gas-chromatographische Charakterisierung organischer Verbindungen. Teil 1: Retentionsindices aliphatischer Halogenide, Alkohole, Aldehyde und Ketone. Helv Chim Acta.

[30] Dunn WJ, Block JH, PR S (1986). Partition coefficient, determination and estimation.

[31] Mannhold R, Poda GI, Ostermann C, Tetko IV (2009). Calculation of molecular lipophilicity: state-of-the-art and comparison of log P methods on more than 96,000 compounds. J Pharm Sci.

[32] Kern S, Fenner K, Singer HP, Schwarzenbach RP, Hollender J (2009). Identification of transformation products of organic contaminants in natural waters by computer-aided prediction and high-resolution mass spectrometry. Environmental Sci Technol.

[33] Bade R, Bijlsma L, Sancho JV, Hernández F (2015). Critical evaluation of a simple retention time predictor based on LogKow as a complementary tool in the identification of emerging contaminants in water. Talanta.

[34] Hogenboom A, Van Leerdam J, de Voogt P (2009). Accurate mass screening and identification of emerging contaminants in environmental samples by liquid chromatography–hybrid linear ion trap Orbitrap mass spectrometry. J Chromatogr A.

[35] Steinbeck C, Han Y, Kuhn S, Horlacher O, Luttmann E, Willighagen E (2003). The chemistry development kit (CDK): an open-source java library for chemo- and bio-informatics. J Chem Inf Comput Sci.

[36] Steinbeck C, Hoppe C, Kuhn S, Floris M, Guha R, Willighagen EL (2006). Recent developments of the chemistry development kit (CDK)—an open-source java library for chemo- and bio-informatics. Curr Pharm Des.

[37] Ulrich N, Schüürmann G, Brack W (2011). Linear solvation energy relationships as classifiers in non-target analysis—a capillary liquid chromatography approach. J Chromatogr A.

[38] Miller TH, Musenga A, Cowan DA, Barron LP (2013). Prediction of chromatographic retention time in high-resolution anti-doping screening data using artificial neural networks. Anal Chem.

[39] Cao M, Fraser K, Huege J, Featonby T, Rasmussen S, Jones C (2015). Predicting retention time in hydrophilic interaction liquid chromatography mass spectrometry and its use for peak annotation in metabolomics. Metabolomics.

[40] Heller SR, McNaught A, Stein S, Tchekhovskoi D, Pletnev IV (2013) InChI—the worldwide chemical structure identifier standard. J Cheminform 5(7). doi:10.1186/1758-2946-5-7 10.1186/1758-2946-5-7 PMC3599061 23343401

[41] Ruttkies C, Strehmel N, Scheel D, Neumann S (2015). Annotation of metabolites from gas chromatography/atmospheric pressure chemical ionization tandem mass spectrometry data using an in silico generated compound database and MetFrag. Rapid Commun Mass Spectrom.

[42] Daylight Chemical Information Systems, Inc. (2016) SMILES—a simplified chemical language. http://www.daylight.com/dayhtml/doc/theory/theory.smiles.html. Accessed 14 Jan 2016

[43] Ridder L, van der Hooft JJJ, Verhoeven S, de Vos RCH, van Schaik R, Vervoort J (2012). Substructure-based annotation of high-resolution multistage MSn spectral trees. Rapid Commun Mass Spectrom.

[44] Daylight Chemical Information Systems, Inc. (2016) SMARTS—a language for describing molecular patterns. http://www.daylight.com/dayhtml/doc/theory/theory.smarts.html. Accessed 14 Jan 2016

[45] Ma Y, Kind T, Yang D, Leon C, Fiehn O (2014). MS2Analyzer: a software for small molecule substructure annotations from accurate tandem mass spectra. Anal Chem.

[46] National Center for Biotechnology Information (2016) PubChem REST Services. https://pubchem.ncbi.nlm.nih.gov/pug_rest/PUG_REST_Tutorial.html. Accessed 14 Jan 2016

[47] Royal Society of Chemistry (2016) ChemSpider MassSpec API. http://www.chemspider.com/MassSpecAPI.asmx. Accessed 14 Jan 2016

[48] Leo AJ (1993). Calculating log Poct from structures. Chem Rev.

[49] Wang R, LL Fu Y (1997). A new atom-additive method for calculating partition coefficients. J Chem Inf Comput Sci.

[50] Cheng T, Zhao Y, Li X, Lin F, Xu Y, Zhang X, Li Y, Wang R, Lai L (2007). Computation of octanol-water partition coefficients by guiding an additive model with knowledge. J Chem Inf Model.

[51] Stravs MA, Schymanski EL, Singer HP, Hollender J (2013). Automatic recalibration and processing of tandem mass spectra using formula annotation. J Mass Spectrom.

[52] Stravs MA, Schymanski EL (2016) RMassBank Package. http://www.bioconductor.org/packages/devel/bioc/html/RMassBank.html. Accessed 14 Jan 2016

[53] Smith CA, Want EJ, O’Maille G, Abagyan R, Siuzdak G (2006). XCMS: processing mass spectrometry data for metabolite profiling using nonlinear peak alignment, matching, and identification. Anal Chem.

[54] Kerber A, Meringer M, Rücker C (2006). CASE via MS: ranking structure candidates by mass spectra. Croat Chem Acta.

[55] Schymanski EL, Meringer M, Brack W (2009). Matching structures to mass spectra using fragmentation patterns: are the results as good as they look?. Anal Chem.

[56] ChemAxon (2016) JChem for Excel 15.7.2700.2799. http://www.chemaxon.com. Accessed 14 Jan 2016

[57] AMBIT (2016) AMBIT Web. https://apps.ideaconsult.net/ambit2/depict. Accessed 14 Jan 2016

