# Supplementary figures and images for: MetFrag relaunched: incorporating strategies beyond in silico fragmentation
Source: J Cheminform. 2016 Jan 29;8:3. doi: 10.1186/s13321-016-0115-9 (PMC4732001; doi:10.1186/s13321-016-0115-9)

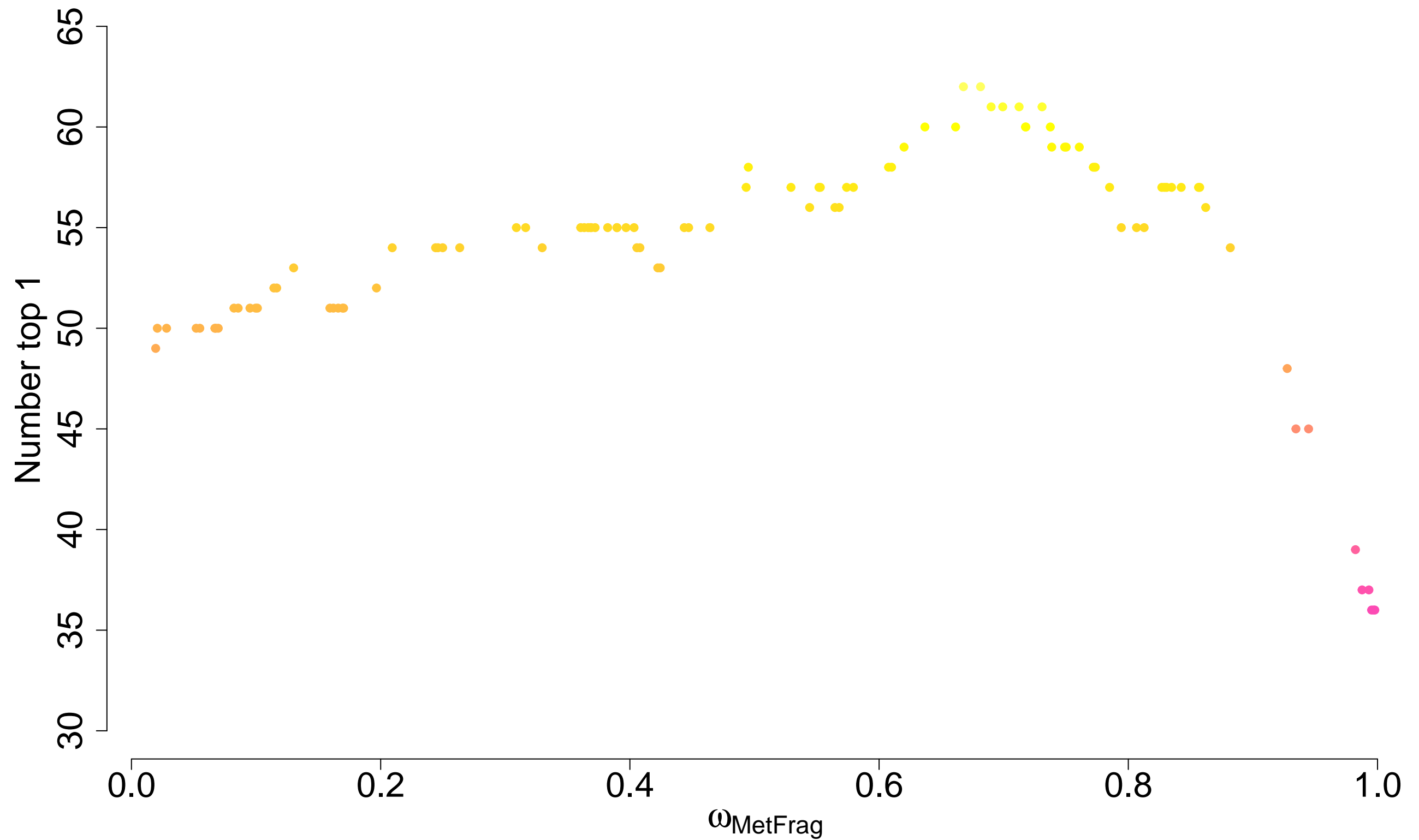

Supplement: Supplementary file 4 — 10.1007/s10898-015-0115-8 Top 1 ranks of MetFrag2.2. combined with CFM--ID This figure shows the distribution of the number of top 1 ranks with different weights (100 drawn randomly between 0 and 1) for MetFrag2.2 and CFM--ID. Lightestyellow dot marks the maximum, 62 top 1 ranks at MetFrag = 0.67 and CFM-ID = 0.33. The red dot at the right marks the minimum, 36 top 1 ranks at MetFrag = 0.997 and CFM-ID = 0.003. The most left dot marks 49 top 1 ranks at MetFrag = 0.02 and CFM-ID = 0.98. [file 13321_2016_115_MOESM4_ESM.pdf]

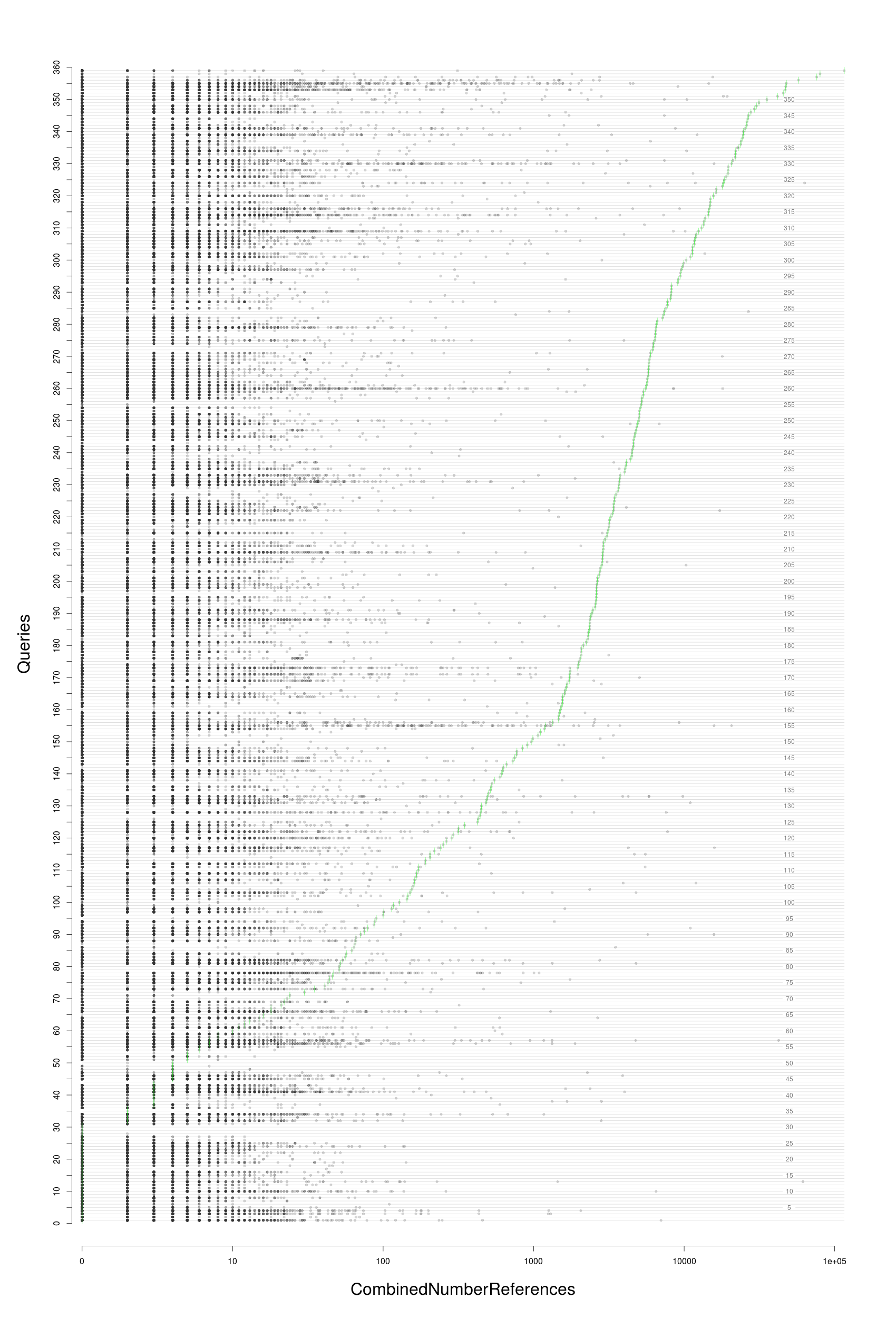

Supplement: Supplementary file 5 — 10.1007/s10898-015-0115-8 Number of patents and PubMed references shown as CombinedReferences retrieved from PubChem for the Orbitrap XL dataset This figure shows the distribution of the number of references and patents for all candidates (marked by black dots) retrieved from PubChem for the 359 (unqiue) correct candidates (marked with green line) and the additional (wrong) candidates retrieved for each query. The queries are sorted by the number of CombinedReferences for the correct candidate, respectively. The intensity of the black dots indicate the number of candidates which overlap at that position. [file 13321_2016_115_MOESM5_ESM.png]

Number of Top 5 hits on 473 MS/MS spectra with PubChem (PubChem XlogP3)  
(Refs = Patents + References)

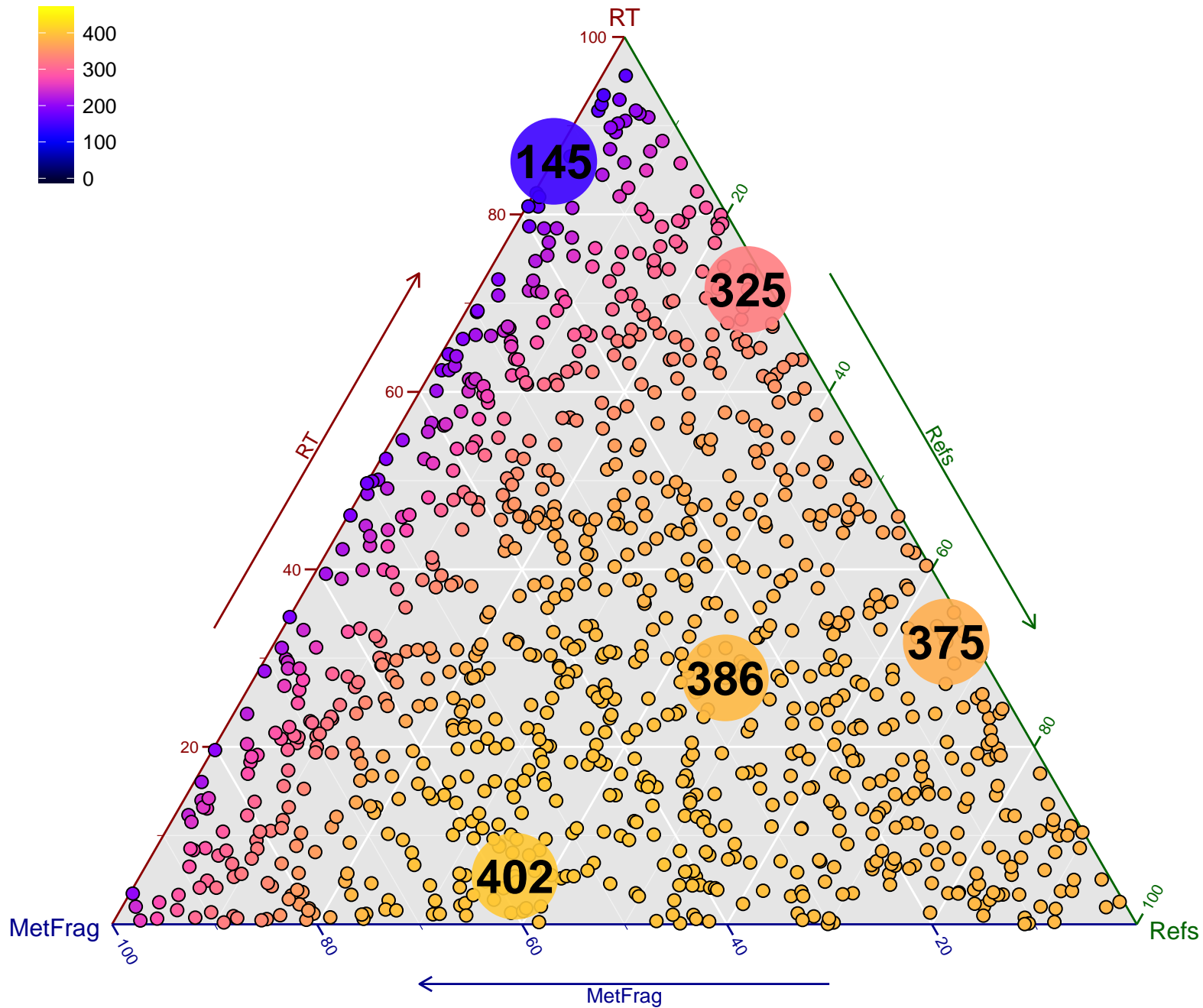

Supplement: Supplementary file 6 — 10.1007/s10898-015-0115-8 Top 5 ranks with PubChem (XlogP3) on the Orbitrap XL Dataset The results were obtained with MetFrag2.2 formula query and the inclusion of patents, references and retention time. Each small dot shows the number of first ranks with a given combination of weights. The larger dots show the best result (402 in the top 5), 90th percentile (386), median (375), 10th percentile (325) and worst result (145). [file 13321_2016_115_MOESM6_ESM.pdf]

Number of Top 5 hits on 473 MS/MS spectra with ChemSpider  
(Refs = ReferenceCount)

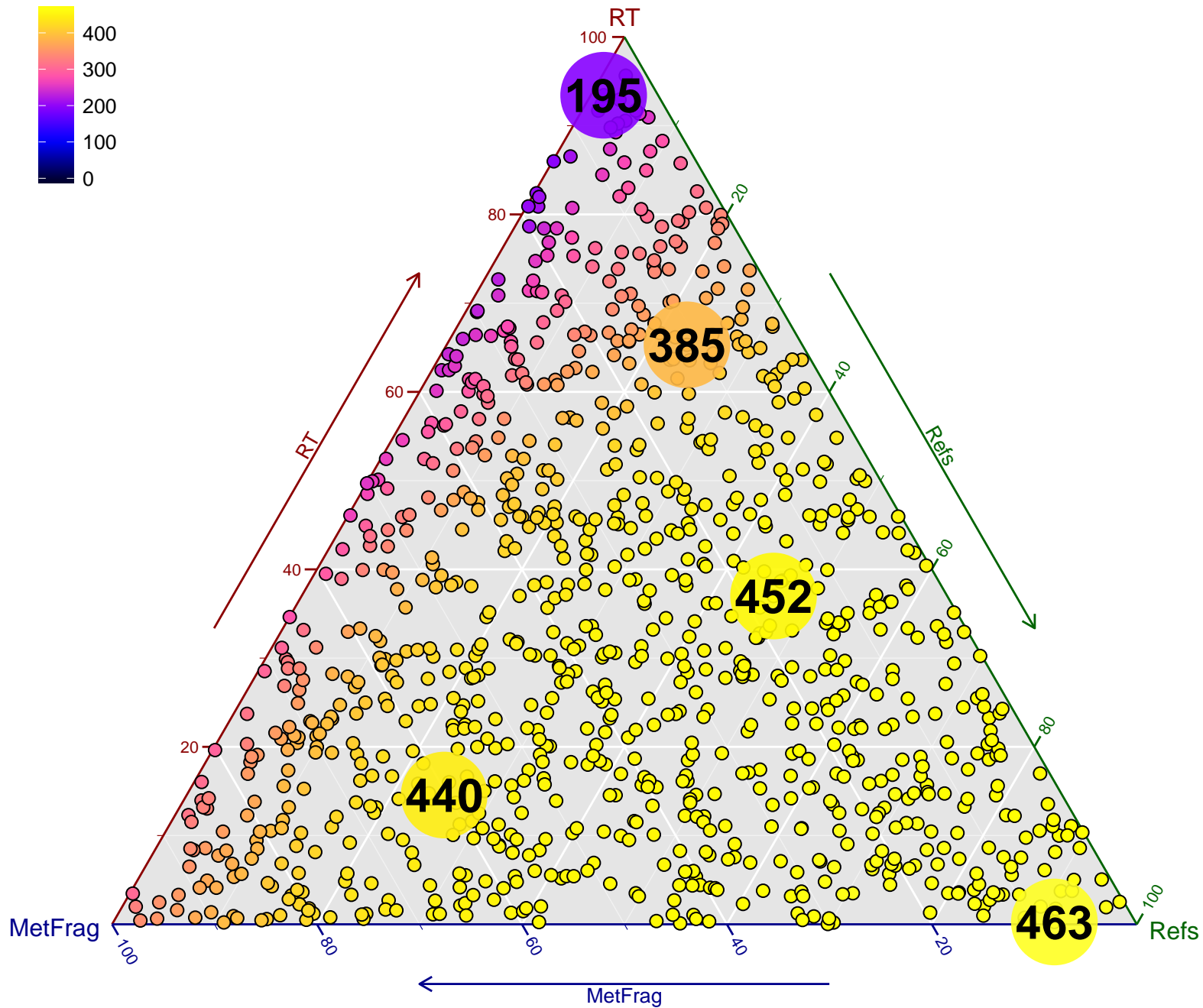

Supplement: Supplementary file 7 — 10.1007/s10898-015-0115-8 Top 5 ranks with ChemSpider on the Orbitrap XL Dataset The results were obtained with MetFrag2.2 formula query and the inclusion of references and retention time. Each small dot shows the number of first ranks with a given combination of weights. The larger dots show the best result (463 in the top 5), 90th percentile (452), median (440), 10th percentile (385) and worst result (195). [file 13321_2016_115_MOESM7_ESM.pdf]

Number of Top 10 hits on 473 MS/MS spectra with PubChem (PubChem XlogP3)  
(Refs = Patents + References)

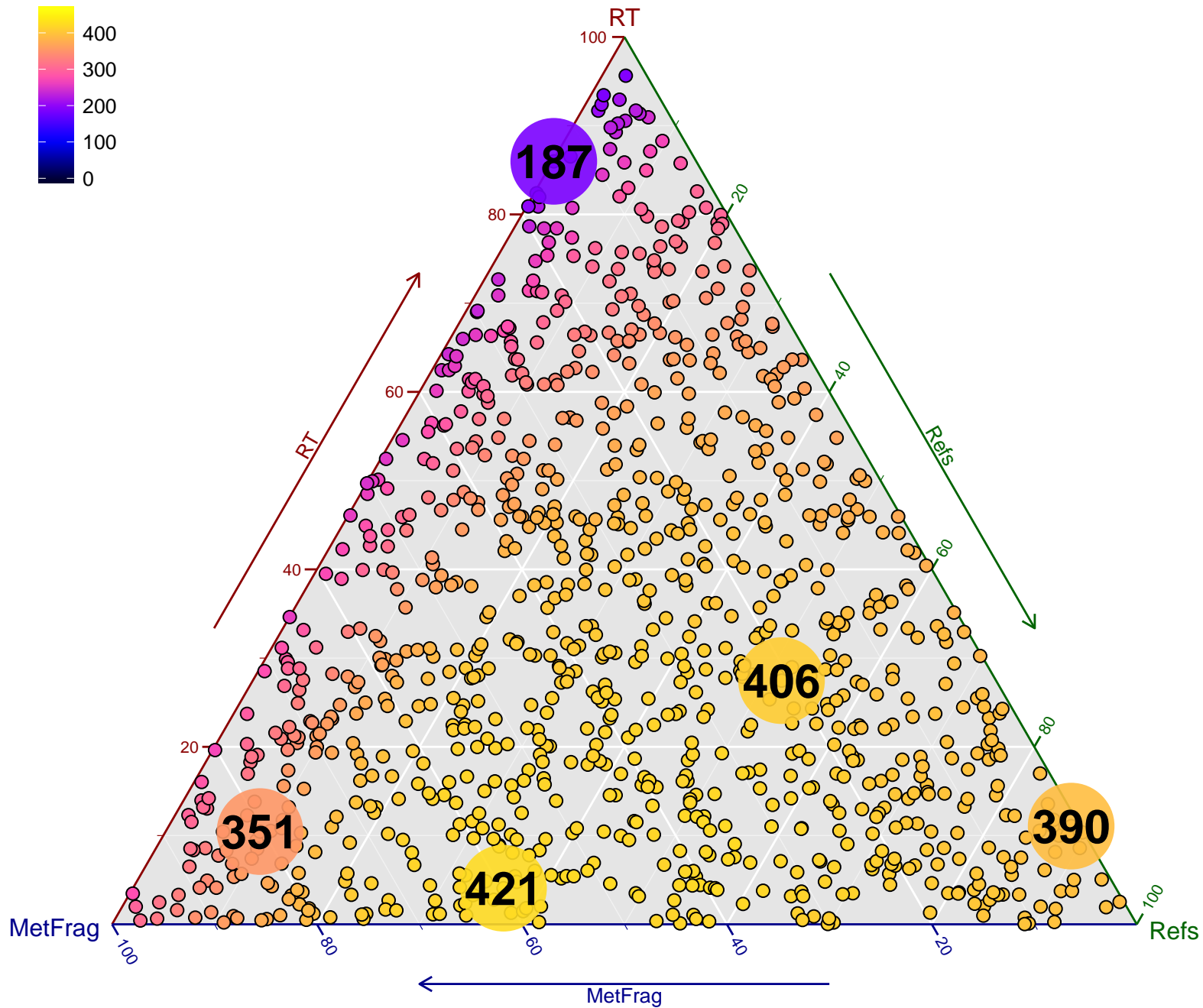

Supplement: Supplementary file 8 — 10.1007/s10898-015-0115-8 Top 10 ranks with PubChem (XlogP3) on the Orbitrap XL Dataset The results were obtained with MetFrag2.2 formula query and the inclusion of patents, references and retention time. Each small dot shows the number of first ranks with a given combination of weights. Each small dot shows the number of first ranks with a given combination of weights. The larger dots show the best result (422 in the top 10), 90th percentile (406), median (391), 10th percentile (351) and worst result (187). [file 13321_2016_115_MOESM8_ESM.pdf]

Number of Top 10 hits on 473 MS/MS spectra with ChemSpider  
(Refs = ReferenceCount)

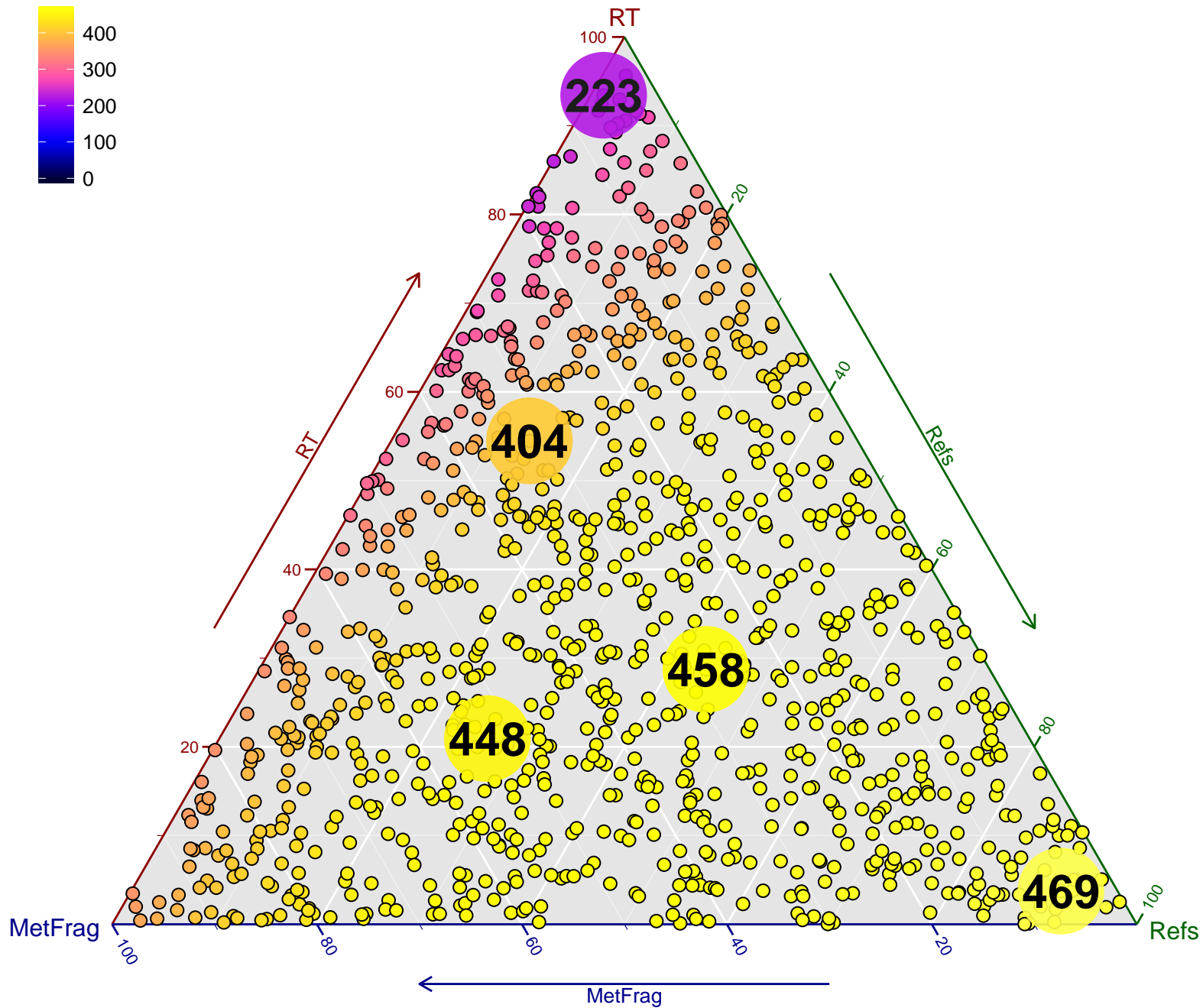

Supplement: Supplementary file 9 — 10.1007/s10898-015-0115-8 Top 10 ranks with ChemSpider on the Orbitrap XL Dataset The results were obtained with MetFrag2.2 formula query and the inclusion of references and retention time. Each small dot shows the number of first ranks with a given combination of weights. The larger dots show the best result (471 in the top 10), 90th percentile (460), median (450), 10th percentile (404) and worst result (223). [file 13321_2016_115_MOESM9_ESM.pdf]

Number of Top 1 hits on 225 MS/MS spectra with PubChem (PubChem XlogP3)  
(Refs = Patents + References)

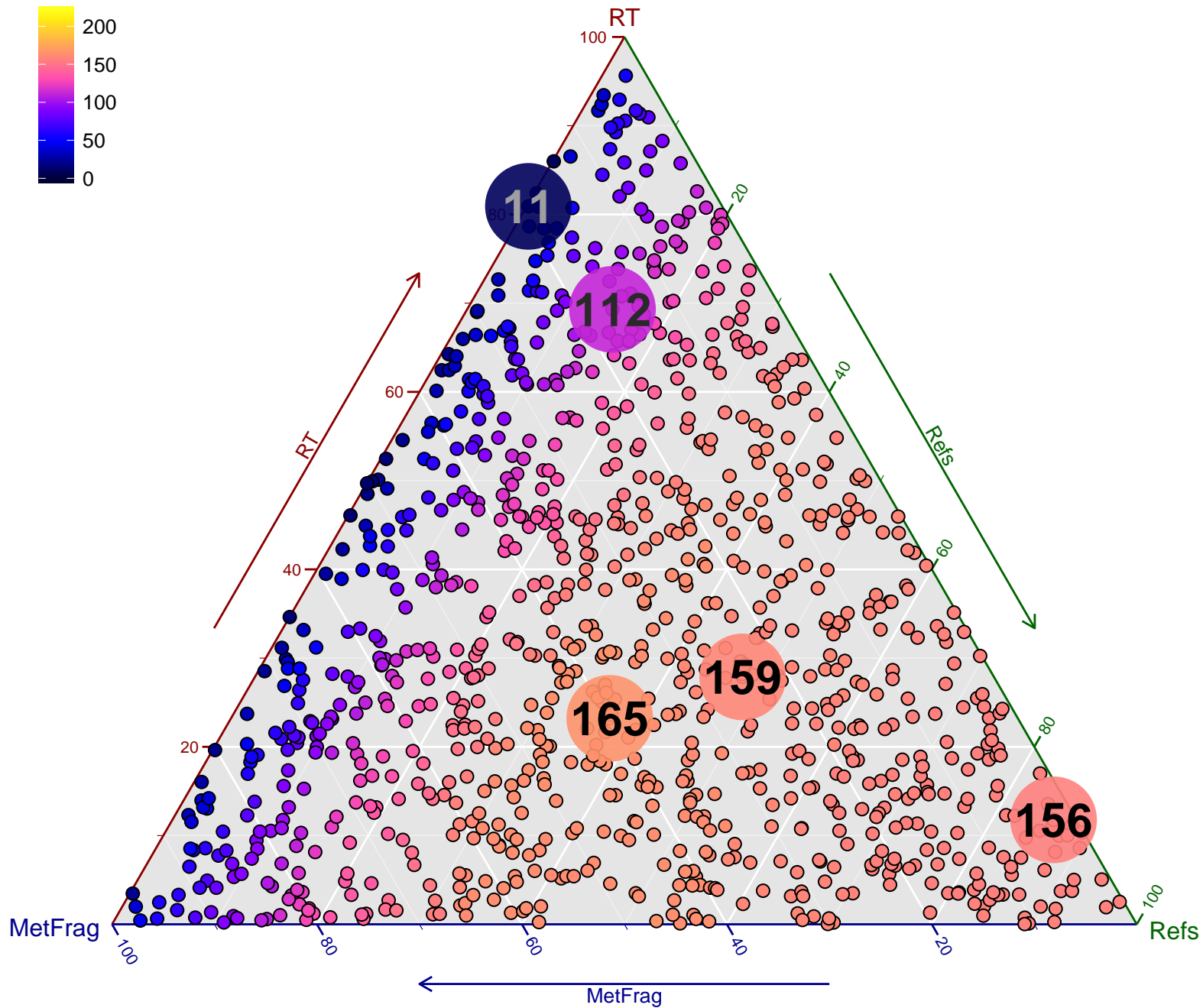

Supplement: Supplementary file 10 — 10.1007/s10898-015-0115-8 Top 1 ranks with PubChem (XlogP3) on the UFZ dataset The results were obtained with MetFrag2.2 formula query and the inclusion of patents, references and retention time. Each small dot shows the number of first ranks with a given combination of weights. The larger dots show the best result (165 in the top 1), 90th percentile (159), median (156), 10th percentile (112) and worst result (11). [file 13321_2016_115_MOESM10_ESM.pdf]

Number of Top 1 hits on 289 MS/MS spectra with PubChem (PubChem XlogP3)  
(Refs = Patents + References)

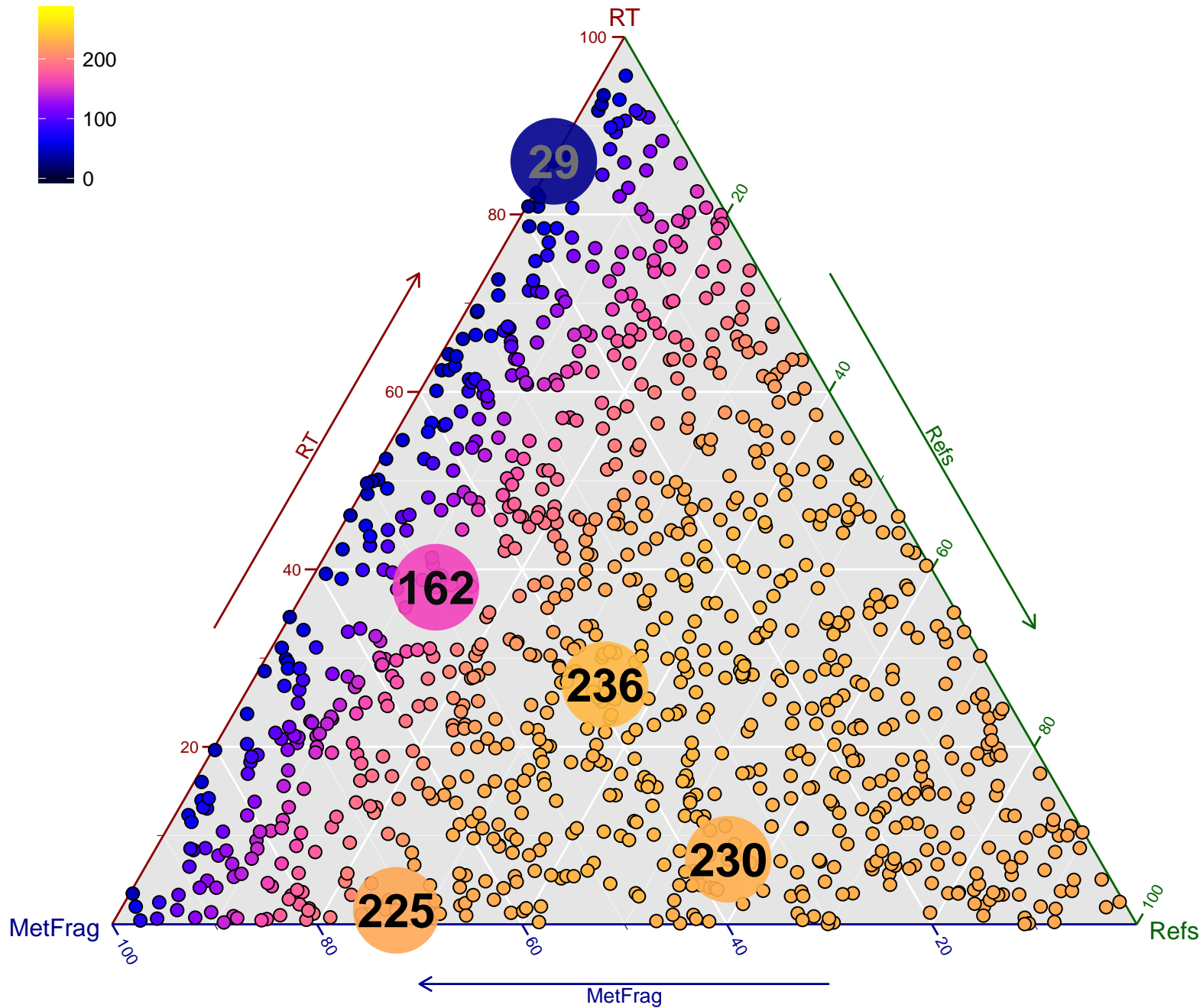

Supplement: Supplementary file 11 — 10.1007/s10898-015-0115-8 Top 1 ranks with PubChem (XlogP3) on the EQex dataset The results were obtained with MetFrag2.2 formula query and the inclusion of patents, references and retention time. Each small dot shows the number of first ranks with a given combination of weights. The larger dots show the best result (236 in the top 1), 90th percentile (230), median (225), 10th percentile (162) and worst result (29). [file 13321_2016_115_MOESM11_ESM.pdf]

Number of Top 1 hits on 310 MS/MS spectra with PubChem (PubChem XlogP3)  
(Refs = Patents + References)

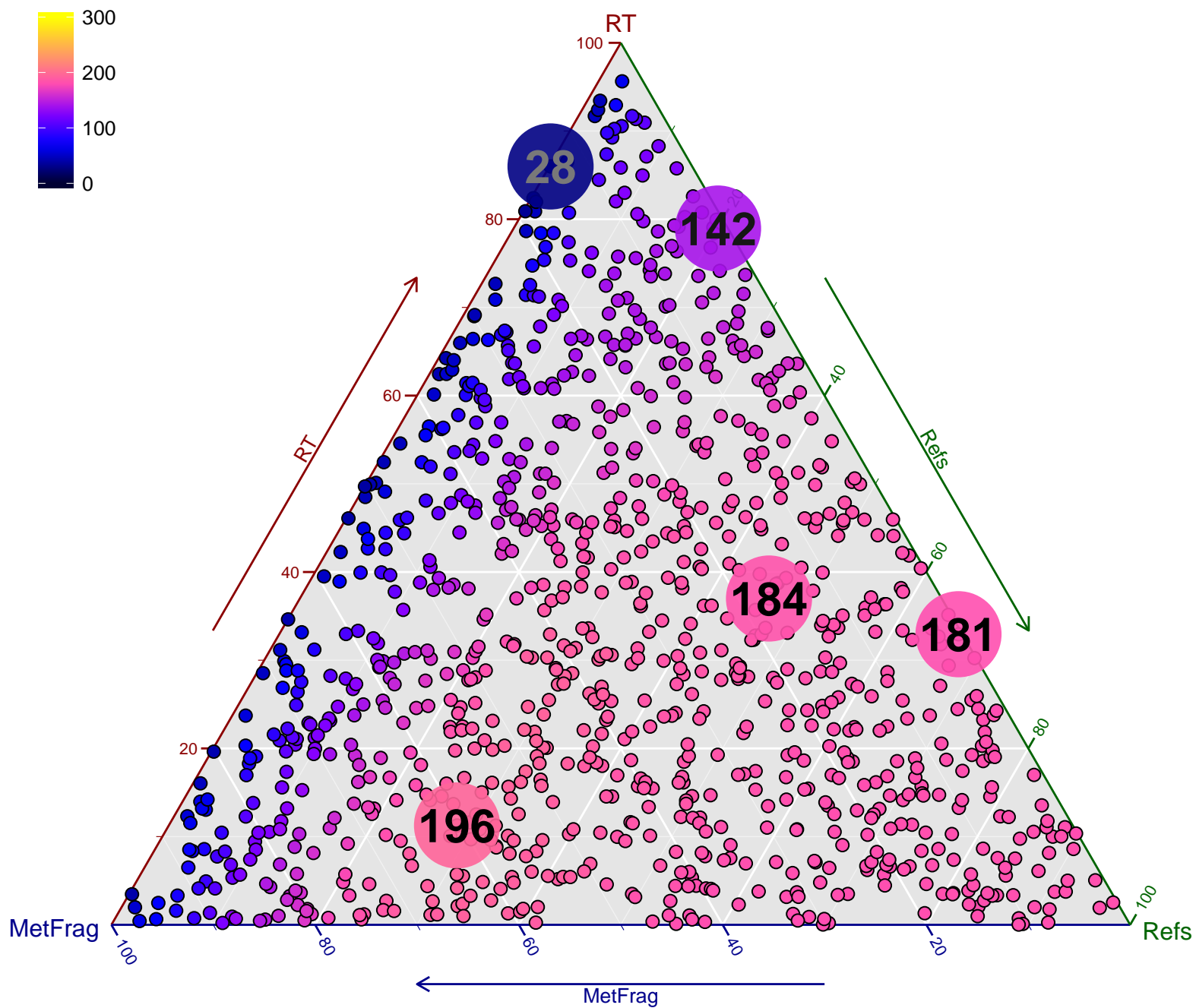

Supplement: Supplementary file 12 — 10.1007/s10898-015-0115-8 Top 1 ranks with PubChem (XlogP3) on the EQexPlus dataset The results were obtained with MetFrag2.2 formula query and the inclusion of patents, references and retention time. Each small dot shows the number of first ranks with a given combination of weights. The larger dots show the best result (196 in the top 1), 90th percentile (184), median (181), 10th percentile (142) and worst result (28). [file 13321_2016_115_MOESM12_ESM.pdf]
